# Early infection-induced natural antibody response

**DOI:** 10.1038/s41598-021-81083-0

**Published:** 2021-01-15

**Authors:** Klara Kubelkova, Tomas Hudcovic, Hana Kozakova, Jaroslav Pejchal, Ales Macela

**Affiliations:** 1 Department of Molecular Pathology and Biology, Faculty of Military Health Sciences, University of Defence, Trebesska 1575, 500 01 Hradec Kralove, Czech Republic; 2 Department of Immunology and Gnotobiology, Institute of Microbiology of the Academy of Sciences of the Czech Republic, Videnska 1083, 142 20 Prague 4, Czech Republic; 3 Department of Toxicology and Military Pharmacy, Faculty of Military Health Sciences, University of Defence, Trebesska 1575, 500 01 Hradec Kralove, Czech Republic

**Keywords:** Infection, Innate immunity, Immunology, Microbiology, Pathogens

## Abstract

There remains to this day a great gap in understanding as to the role of B cells and their products—antibodies and cytokines—in mediating the protective response to *Francisella tularensis*, a Gram-negative coccobacillus belonging to the group of facultative intracellular bacterial pathogens. We previously have demonstrated that *Francisella* interacts directly with peritoneal B-1a cells. Here, we demonstrate that, as early as 12 h postinfection, germ-free mice infected with *Francisella tularensis* produce infection-induced antibody clones reacting with *Francisella tularensis* proteins having orthologs or analogs in eukaryotic cells. Production of some individual clones was limited in time and was influenced by virulence of the *Francisella* strain used. The phylogenetically stabilized defense mechanism can utilize these early infection-induced antibodies both to recognize components of the invading pathogens and to eliminate molecular residues of infection-damaged self cells.

## Introduction

*Francisella tularensis* is a Gram-negative coccobacillus belonging to the group of facultative intracellular bacterial pathogens proliferating in phagocytic cells, including neutrophils. Several subspecies of *F. tularensis* cause serious tularemia infection in both animals and humans. A safe and effective vaccine continues to be sought to protect against respiratory infection with hypervirulent *F. tularensis* subsp. *tularensis*^1^. One of the reasons we do not yet have a suitable vaccine relates to the considerable gaps in our knowledge of the body’s defense mechanisms against this dangerous pathogen.

The immune mechanisms contributing to the expression of protective immune response against *F. tularensis* remain a matter of debate. The theories and views as to the roles of individual cell types and subtypes in generating protective immune response are as diverse as are the infection models used to reveal their function. Originally, the T cell-mediated immune processes based on IFN-γ-dependent activation of macrophages were regarded as constituting the decisive factor in host defense against tularemia^2–5^. Although we have intensively studied macrophages as the primary hosts for *Francisella* replication^6–10^, the evaluation of their role in protection remains to this day dependent on the experimental approach taken and virulence of the *Francisella* strains used^11,12^. The contribution of polymorphonuclear leukocytes to the limitation and/or dissemination of infection around the body is similarly ambiguous^13–15^. The currently prevailing opinion is that neutrophils create detrimental effects by participating in tissue destruction following *F. tularensis* infection and thus contribute to pathogenic processes^16^. The primary interaction of dendritic cells with *F. tularensis*, which, as in other phagocytic cells, escapes from the phagosome and proliferates within the dendritic cell cytosol, ends with cytokine-producing cells that have not been sufficiently stimulated and therefore have limited capacity to influence the protective immune response^17,18^. As in the case of macrophages, however, the signaling pathways of dendritic cells are differentially regulated by virulent and attenuated strains of *Francisella*^19^.

Since the 1970s, cellular immunity to tularemia has been regarded as an immunologically specific component of protective response^20–23^. Logically, the T lymphocytes have become the target of attention^24–26^. The alpha/beta CD4 + and CD8 + lymphocytes are equally effective in resolving infection by primary sublethal *F. tularensis* attenuated live vaccine strain (LVS) and establishing protective response to secondary lethal challenge^27^. Moreover, gamma/delta T cells^28–30^ and such other rare T cell populations as, for example, CD4^−^CD8^−^CD3^+^alpha/beta^+^ gamma/delta^−^ NK1.1^−^ T cells^31^ can participate in the control of intracellular bacterium proliferation.

There remains to this day, a great gap in our knowledge as to the role of B cells in mediating protective response to *F. tularensis* infection. The basic function of B lymphocytes within the humoral branch of the adaptive immune system is to generate a spectrum of specific antibodies. According to the prevailing notion, however, the specific antibodies are not a decisive component of protective immunity. Early studies on lymphocyte response to the vaccine strain of *F. tularensis* demonstrated response of both T and B cell populations^32^. Gradually, it was shown that strong early protection, detectable on the third day after infection, is highly dependent on B cells^33^. Limitation of bacterial growth was in this case dependent on IFN-γ and probably independent of the antibodies. Subsequently, a regulatory role of B cells in controlling the secondary immune response to *F. tularensis* LVS infection that is independent of antibody production was demonstrated. The influence of B cells in controlling the classical primary response to *F. tularensis* was marginal^34^. When *F. novicida* was used instead of *F. tularensis* LVS, however, both primary and secondary murine infections were critically dependent on the B cells^35^. Both in vitro and in vivo experiments later proved that murine and human B cell lines, as well as murine peritoneal B cell subsets, are targets of *F. tularensis* invasion^36^. The internalization process in B-1a cells was observed to be dependent on functional surface B cell receptors alone. In B-1b and B-2 cells, by contrast, these receptors needed co-signaling from the co-receptor CR1/2 to initiate *F. tularensis* engulfment^37^. Infected B cells were activated and produced a range of cytokines, including IFN-γ, IL-12, and TNF-α^38^.

An early attempt to resolve the controversial role of humoral immunity in protecting against *F. tularensis* infection utilized the passive transfer of immune sera collected from human recipients of the live tularemia vaccine to naïve mice. Pooled immune sera were shown to protect mice fully against a lethal challenge with the LVS strain^39^. Contributions of specific antibodies to protection against *F. tularensis* infection were then repeatedly demonstrated in several infection models^40–45^. The immune sera used in the aforementioned studies were isolated from infected individuals during either the acute or convalescent phase of infection. In the course of our studies on the mutual interaction of *F. tularensis* with B cells^36,37,45,46^, we inadvertently identified that the antibody clones reacted with the *F. tularensis* proteins ATP synthase α chain, elongation factor TU, universal stress protein, ABC transporter, putative outer membrane protein OmpH, peroxidase/catalase, and chaperone protein DnaK in murine sera isolated 12 h postinfection from specific-pathogen free (SPF) Balb/c mice. These specificities were not detected in control sera from naïve SPF mice. To determine whether these clones are cross-reacting antibodies in sera of SPF mice or if there are natural antibodies induced by infection, we used germ-free (GF) mice for further study. Germ-free mice, unlike SPF mice, are not attacked by internal or external bacteria, so the possibility for cross-reactivity of infection-induced antibody clones is limited^47,48^.

Here, we demonstrate that GF mice infected with *F. tularensis* produce natural antibody clones reacting with 87 *F. tularensis* proteins, of which 63 antibody specificities are targeted to proteins having orthologs or analogs in eukaryotic cells. Production of some individual clones was limited in time and was influenced by virulence of the *Francisella* strain used. This early infection-induced antibody production might be a phylogenetically stabilized defense mechanism both to recognize the components of the invading pathogens and to eliminate the molecular residues of infection-damaged self cells.

## Results

### *F. tularensis* elicits early robust total serum antibody response

**Figure 1:**
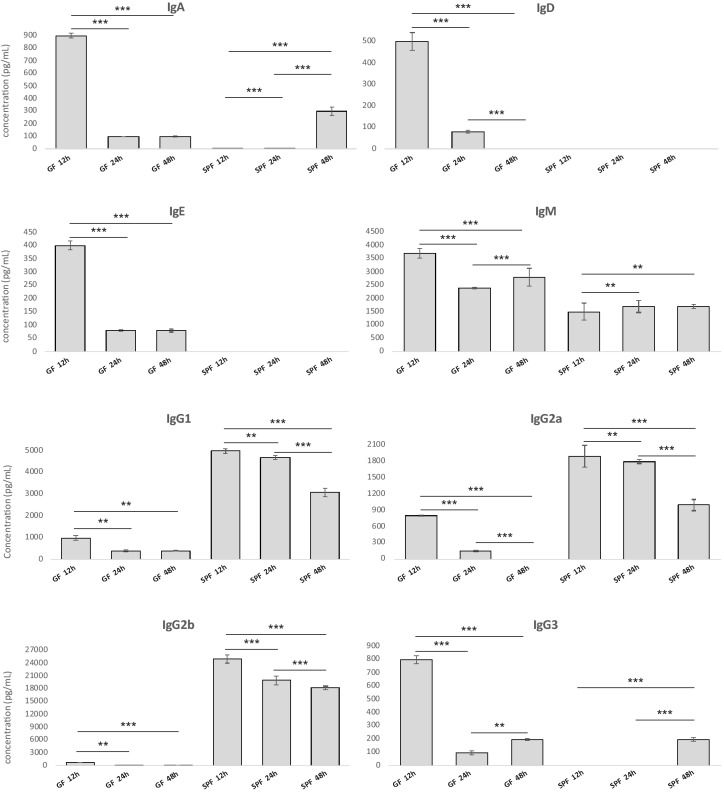
Isotypes of early infection-induced antibodies and their kinetics in Balb/c GF mice sera and Balb/c SPF mice sera obtained 12, 24, and 48 h after *F. tularensis* FSC200 intraperitoneal infection, dose of 10^2^ CFU in total volume of 0.2 mL of saline. The immunoglobulin subclasses of serum samples were determined using a Quantibody mouse immunoglobulin isotype array while following the manufacturer’s instructions. The antibody isotypes compositions of Balb/c SPF and GF murine sera obtained from naïve mice without any bacterial colonization were very low and are not shown in these graphs. The basal individual antibody isotypes levels for GF mice were significantly lower in comparison with the corresponding isotypes levels for SPF mice (data not shown). The Quantibody mouse immunoglobulin isotype arrays were performed in biological triplicates (individual sera) for all time intervals and were independently repeated at least three times. Reference isotypes are presented. Values are expressed as mean ± standard deviation (SD) and analyzed for significance using Student’s two-tailed *t*-test. Differences were considered statistically significant at **p < 0.01 and *p < 0.05 and are denoted by asterisks.

Our initial finding of early antibody production determined by ELISA in SPF mice after *F. tularensis* infection allowed for two interpretations. This reflected either a reaction of cross-reacting pre-existing antibody clones or the rapid natural response of innate B cell subsets. For this reason, we used the GF mice for further experiments and we compared their antibody response with the response of SPF mice. GF animals have no pre-existing contacts with bacteria whereas SPF mice have a natural, pre-existing microbiota, including many non-*Francisella* species. To determine the kinetics of early antibody response to infection, SPF and GF Balb/c mice were infected with *F. tularensis* subsp. *holarctica* FSC200. To ensure direct interaction of antibody-producing cells with bacteria, we used intraperitoneal infection, where direct interaction of individual B cell subtypes is possible without prior processing of the immunogen by antigen-presenting cells. Moreover, the peritoneal cavity provides a suitable model for the study of B cells because of the presence there of a unique peritoneal cavity-resident B cell subset known as B1a cells, in addition to B1b cells and conventional B2 cells. After 12, 24, and 48 h of colonization, total serum antibody levels were measured in each group of mice (Fig. 1). The antibody isotypes composition of Balb/c SPF and GF murine sera obtained from naïve mice without any bacterial colonization were very low and therefore are not shown in this figure. The basal individual antibody isotypes levels for GF mice were significantly lower in comparison with the corresponding isotypes levels for SPF mice. A lower level of circulating IgG1 and IgG2b was observed in normal uninfected GF mice when compared with uninfected SPF mice (Supplementary Fig. S1). A higher level of light kappa and lambda chains in analyzed SPF sera, however, are probably associated with higher content of antibodies in the SPF sera in both normal and infected mice (Supplementary Fig. S2).

The infection of GF as well as SPF mice with *F. tularensis* confirmed that infection induced the early antibody response as soon as 12 h post infection in both mice strains (Fig. 1). The infection with *F. tularensis* significantly increased the levels of all antibody isotypes in GF sera and in sera of SPF mice with the exceptions of IgD and IgE. The IgG1 and IgG2b isotypes dominated in sera of SPF mice, while IgM, IgA, and IgG3 predominated in GF mice. The kinetics of individual antibody isotypes 48 h post infection suggest the infection induces an immediate humoral stress response that is dominantly produced by early-induced natural antibodies.

**Figure 2:**
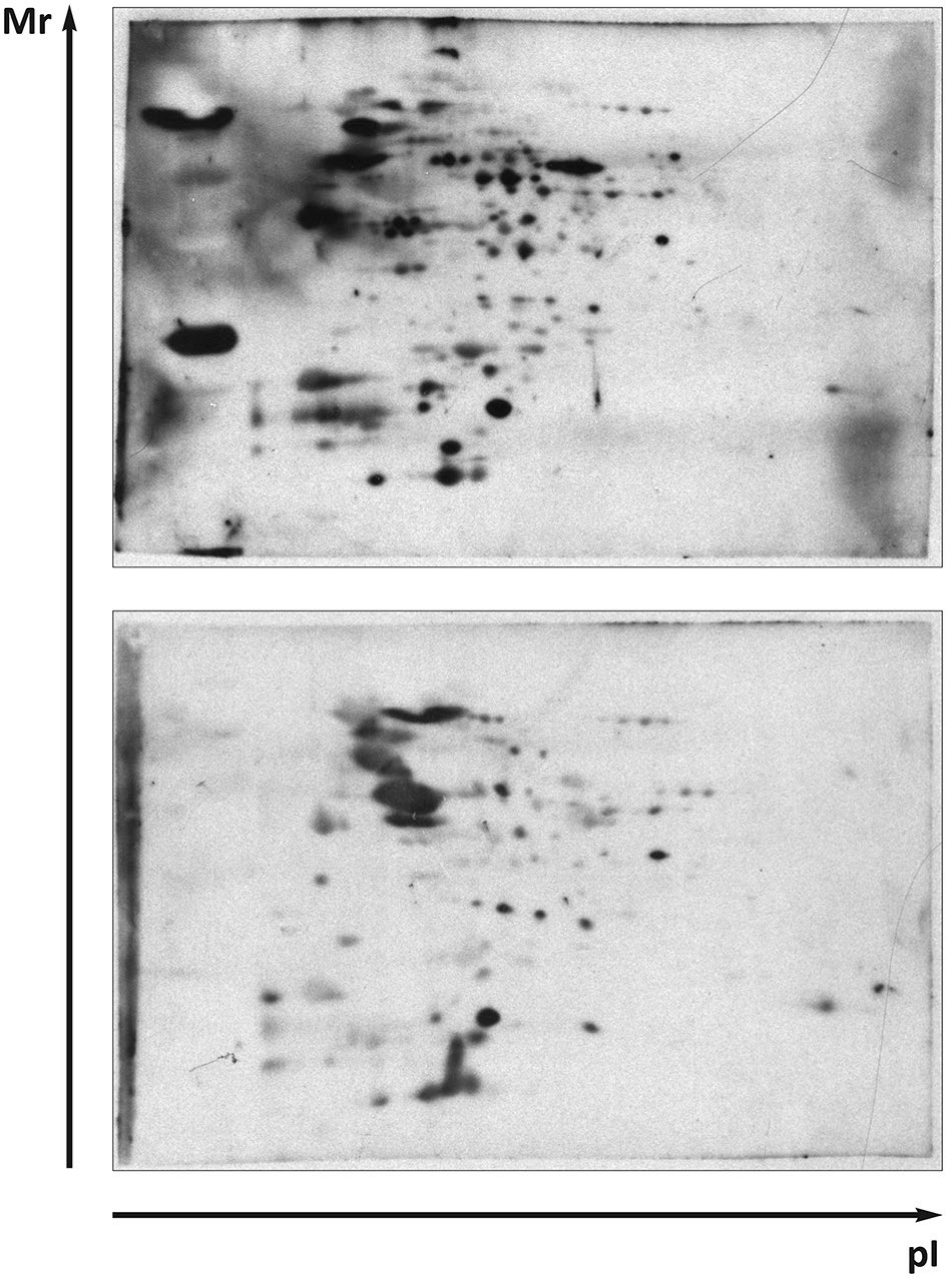
Western blot analysis to evaluate *F. tularensis* FSC200 whole-cell lysate protein targets recognized by sera from *F. tularensis* FSC200 infected Balb/c mice and detected by chemiluminescence. The 2-D reference immunoblots demonstrated the reaction of antibody clones in murine sera obtained from Balb/c GF (upper) and SPF (bottom) mice at 12 h post infection. A total 200 μg of protein was separated by immobilized pH gradient (IPG) strips (3–10) and 12% (w/v) SDS-PAGE gels. Polyclonal peroxidase-conjugated goat anti-mouse IgG antibody was used for secondary antibody detection. All experiments were performed in biological triplicates (individual sera) for all time intervals and were independently repeated at least three times.

Early robust IgM response occurred only after the infection of GF mice with *F. tularensis* subsp. *holarctica* FSC200 at all monitored time points. The IgM antibodies constitute the major component of the natural antibodies and should also be the first class of antibodies produced during early antibody response to infection. Our results demonstrate that this corresponds only to the response of GF mice, as the dominant isotype of early infection-induced antibodies of SPF mice are of IgG2b isotype. Assessed globally, the serum IgM and IgG antibodies were barely detectable in both GF and SPF mice before *Francisella* infection. The early humoral response of GF mice to infection is more intense, however, than is that of SPF mice 12 h post infection (Fig. 2).

The increased level of infection-induced IgM antibody isotype in GF mice at 12 h postinfection and other time intervals in comparison with the levels of IgM isotype in sera of infected SPF mice may be caused by there being a different spectrum of IgM antibody clones (specificities) that reacted with different *Francisella* proteins (Supplementary Fig. S3).

### Molecular targets of the early produced antibodies

**Figure 3:**
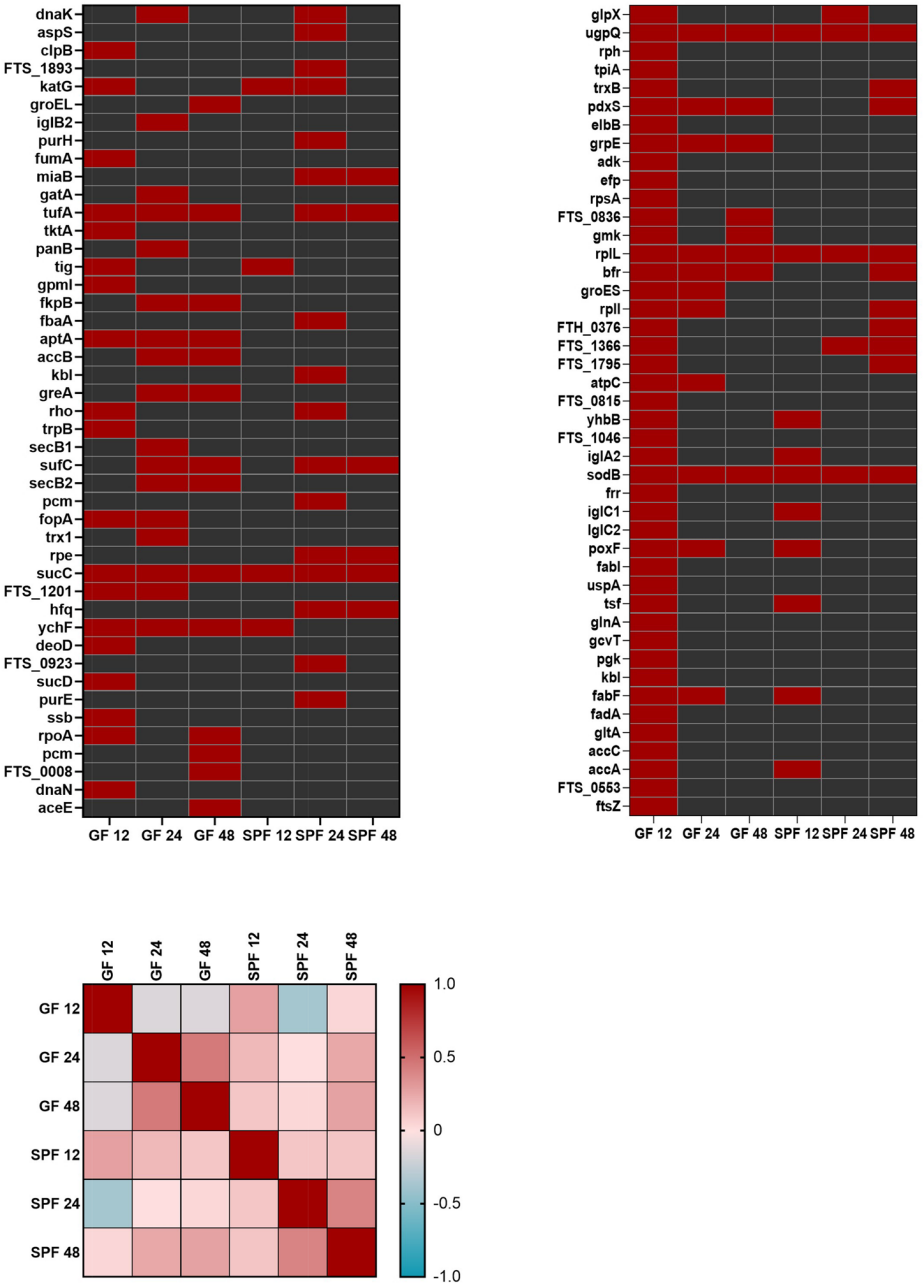
Overview of the *Francisella* proteins identified by mouse sera obtained from Balb/c GF and SPF murine sera collected at 12, 24, and 48 h after *F. tularensis* infection. Similarity analysis used the Pearson curve-based correlation expressed by GraphPad Prism.

**Figure 4:**
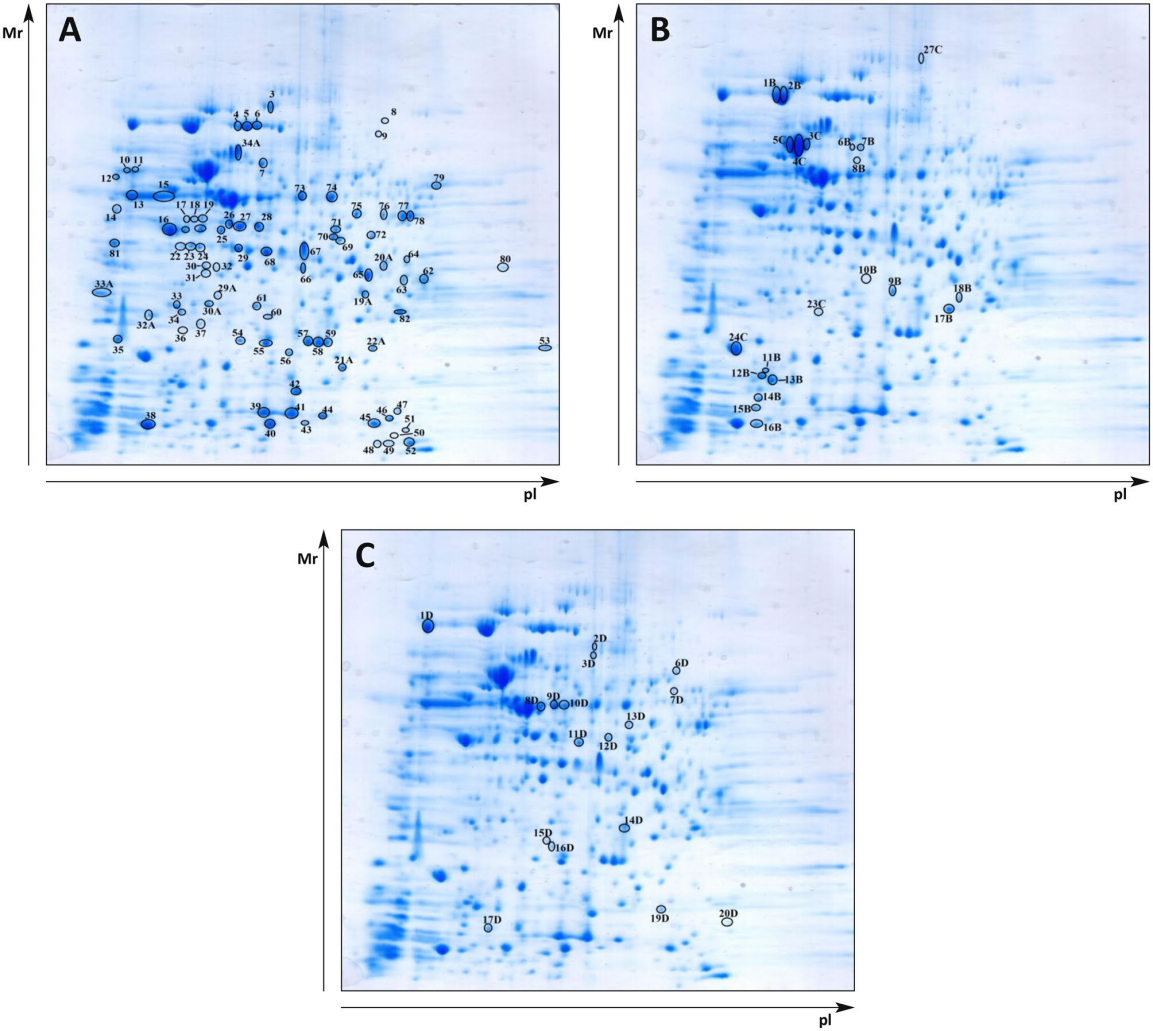
The 2-D reference gels of *F. tularensis* FSC200 whole-cell lysate protein targets, which are recognized by sera from *F. tularensis* FSC200 infected Balb/c GF mice. (**a**) The targets of Balb/c GF mice antibody response at 12 h post infection, (**b**) the depicted unique targets of Balb/c GF mice antibody response at 24 and 48 h post infection, (**c**) the depicted unique antibody response of Balb/c SPF mice at 24 and 48 h post infection. A total 150 μg of protein was separated by immobilized pH gradient (IPG) strips (3–10) and 12% (w/v) SDS-PAGE gels. Identified immunogenic protein spots were numbered in preparative 2-D SDS-PAGE gels, excised for MS/MS analysis, corresponding to the proteins in Table S1 (designated letters and numbers of protein spots were used only for internal purposes and do not necessarily imply the same identity of the labeled proteins). Polyclonal peroxidase-conjugated goat anti-mouse IgG antibody was used for secondary antibody detection. All experiments were performed in biological triplicates (individual sera) for all time intervals and were independently repeated at least three times.

*Francisella tularensis* subs. *holarctica* FSC200 whole-cell lysate was separated by 2-DE using broad range IPG strips of pH 3–10. To validate reproducibility of the separation technique, two independent whole-cell lysates were prepared. Reference silver-stained gel was prepared. Other gels were electroblotted onto polyvinylidene difluoride membrane. Immunoblotting was subsequently performed with mice tularemic sera from SPF and GF mice. Biological triplicates of SPF and GF sera were prepared at selected time points and immunoblotting experiments were performed twice with each serum. Normal murine SPF and GF serum was prepared from blood of Balb/c mice without exposure to any *F. tularensis* strain. Coomassie-stained 2-DE gels were then prepared to excise the selected spots that were digested and the reactivities on bacterial extracts were determined. The mass spectrometry approach successfully assessed in total 87 different *F. tularensis* proteins that reacted with the sera obtained from mice at 12, 24, and 48 h post infection (Fig. 3). A similarity analysis demonstrated that the proteins reacted according to the model (GF versus SPF) that was used and the time intervals when the sera were obtained after infection. The dominant immunoreactive protein spots of *F. tularensis* FSC200 detected on at least three immunoblots are shown on the reference 2-D protein map (Fig. 4) and the proteins are summarized in Supplementary Table S1. A total 50 immunoreactive *Francisella* proteins were unique targets of GF murine sera with no appearance of interaction with SPF sera isolated during the intervals of innate immune response to *F. tularensis* infection (see Fig. 3 and Supplementary Tab. S1).

A number of immunoreactive proteins identified in this study already have been described using different infection models, including human tularemia at the stage of adaptive immune response (see references for Supplementary Table S1). Some studies were conducted to identify and characterize the virulence factors that *F. tularensis* uses to infect a wide variety of hosts, induce disease, and evade immune defense mechanisms. Some of them are listed as immunoreactive proteins. Sera isolated from GF as well as SPF mice during very early time points (i.e., 12, 24, and 48 h) postinfection reacted also with several virulence factors already identified. In this respect, the robust reactions to *Francisella* virulence factors were mainly observed using GF murine serum obtained 12 h after intraperitoneal inoculation by bacteria.

In this study, 19 *F. tularensis* proteins that were recognized by the early infection-induced antibody clones had already been recognized as potential virulence factors with specified function and importance. However, if *F. tularensis* survives in arthropods, fresh water amoeba, and mammals, where it has both intracellular and extracellular phases, it could be reasonable to expect changes in expression of (virulence) factors enabling *Francisella* to survive in these various environments. For these reasons, denomination of *Francisella* virulence factors is debatable^49–53^.

Namely, these are fructose-1,6-bisphosphatase (FTS_1656), chaperone ClpB (FTS_0084), intracellular growth locus A protein (FTS_0097/1125), intracellular growth locus C protein (FTS_0099/1127), superoxide dismutase (FTS_1747), peroxidase/catalase (FTS_1471), host factor I for bacteriophage Q beta replication (FTS_0889), heat shock protein GrpE (FTS_1166), chaperone protein DnaK (FTS_1167), co-chaperonin GroES (FTS_1671), OmpA family protein (FTS_1295), fumarate hydratase (FTS_0527), succinyl-CoA synthetase subunit beta (FTS_1517), succinyl-CoA synthetase subunit alpha (FTS_1518), DNA-directed RNA polymerase subunit alpha (FTS_0258), transcription elongation factor GreA (FTS_1440), glycerophosphoryl diester phosphodiesterase (FTS_1476), 30S ribosomal protein S1 (FTS_1862), bacterioferritin (FTS_0617), and trigger factor (FTS_0882). Moreover, 2 proteins, namely OmpA family protein (FTS_1295) and glycerophosphoryl diester phosphodiesterase (FTS_1476), were annotated as outer membrane associated proteins that might play an important role in immunity during host–pathogen interaction.

The early *Francisella*-induced antibodies reacted also with SodB (FTS_1747), KatG (FTS_1471), DnaK (FTS_1167), and GroES (FTS_1671), which are proteins that already have been reported to be released or secreted by *F. tularensis* but the mechanisms for their secretion remain unclear. In addition, we identified 6 immunoreactive proteins annotated with signal peptide according to SignalP 5.0 software, namely KatG (FTS_1471), OmpA (FTS_1295), GTP-dependent nucleic acid-binding protein EngD (FTS_0935), Omp protein of unknown function (FTS_0008), glycerophosphoryl diester phosphodiesterase (FTS_1476), and hypothetical protein (FTS_0815). All the mentioned proteins are listed along with their characteristics in Supplementary Table S1.

**Figure 5:**
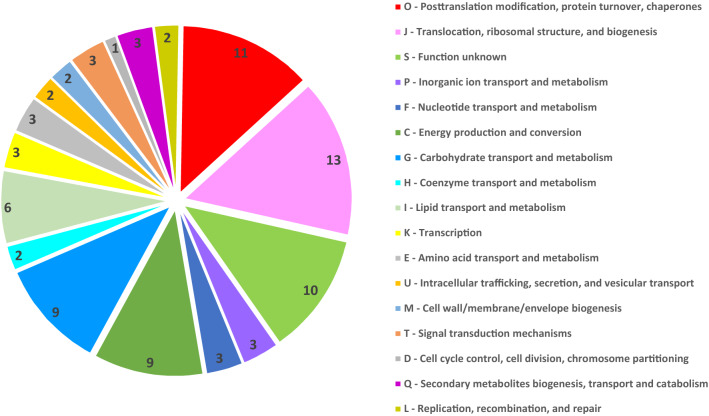
Categorization of infection induced antibody response protein targets according to Clusters of Orthologous Groups (COGs) characterizing the functional as well as topographical characteristics of identified protein groups as predicted by EggNOG v. 5.0.0. All identified protein targets by immune sera of both mouse models and all time intervals tested are summarized.

**Figure 6:**
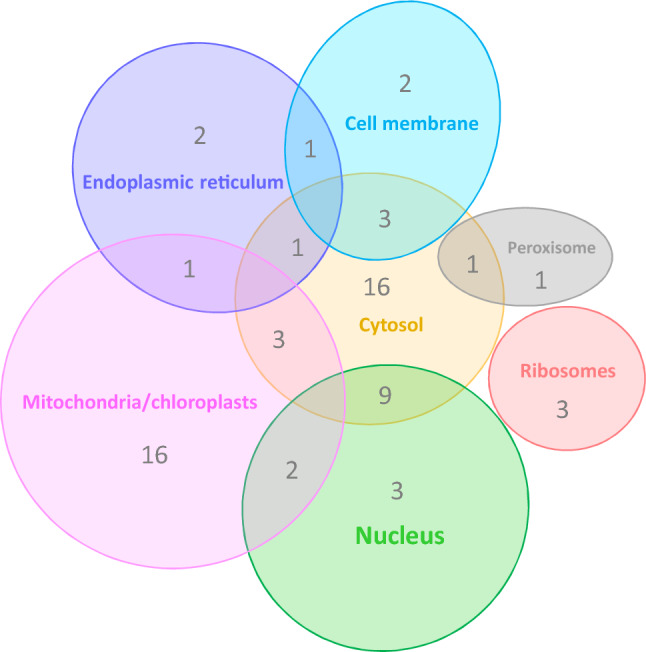
Venn diagram representing the eukaryotic cell compartments within which are summarized orthologs or analogs of all *F. tularensis* proteins reactive with early infection-induced antibody clones. The numbers within sectors represent a set of proteins located at a given cellular compartment. Data on cell localization were taken from the UniProtKB database.

All identified proteins reacting with the sera from *Francisella*-infected GF and SPF mice were sorted into Clusters of Orthologous Groups (COG) functional categories (Fig. 5). Two dominant COGs relate to biogenesis at the stage of protein post-translational modification and chaperon-accompanied protein translocation. Sixty-three of the 87 identified antibody clones reacted with bacterial proteins that have orthologs or analogs in eukaryotic cells. The remaining 24 prokaryotic targets are characteristic for *F. tularensis*, of which 7 are hypothetical proteins. The identities of those proteins were determined according to the UniProtKB database. Among the eukaryotic analogs or orthologs, mitochondrial and cytosolic proteins were found to be the dominant targets of early *Francisella*-induced antibodies (Fig. 6). The reactivities of all SPF and GF murine sera obtained at the selected time points are described in Supplementary Table S1. Of 50 unique GF antibody specificities, the serum of GF mice contains 12 with no eukaryotic orthologs (Table S2). These antibody specificities have protein targets that, according to the Clusters of Orthologous Groups categorization, are involved on the one hand in amino acid metabolism and transport, protein intracellular trafficking and secretion, and membrane biogenesis, and on the other hand in cell cycle control and replication and repair (see Fig. 6).

### *Francisella*-induced early unspecific antibody response

The sera from Balb/c mice infected with *F. tularensis* subs. *holarctica* FSC200 obtained in early 12 h interval after infection reacted with different sets of *F. tularensis* subsp. *holarctica* FSC200 and *F. tularensis* LVS proteins used in the form of whole-cell lysates for immunoblotting. The intensity of reaction corresponded to virulence of the bacterial strain used for infection. The early humoral response of mice to *F. tularensis* subs. *holarctica* FSC200 is more intense in terms of both the quantity of antibodies in serum and the spectrum of recognized targets when compared with LVS counterparts. In parallel with immunoblotting of sera obtained from GF and SPF mice infected by *F. tularensis* against *F. tularensis* whole-cell lysate proteins we used as targets also the whole-cell lysates of *Klebsiella pneumonia* (clinical isolate) and *Pseudomonas aeruginosa* CCM 3955. Both bacteria are conditionally pathogenic and can incidentally colonize the airways. The reaction of early *Francisella*-induced antibodies on these targets was weaker than was the response against *F. tularensis* whole-cell lysate, but it was still very significant (Supplementary Fig. S4). The reactions of protein targets of both alternative microbes with the sera obtained from *F. tularensis*-infected GF mice were more intense than was the response of sera obtained from SPF mice. The GF control sera failed to react with *Klebsiella pneumoniae* and *Pseudomonas aeruginosa* proteins. The control sera obtained from SPF mice reacted, but only slightly, with the proteins of both bacteria. The target proteins for the sera from *Francisella*-infected mice were not identified because we did not search for them.

If the sera from both GF and SPF *Francisella*-infected mice reacted with proteins of unrelated bacteria we tested also the response of these sera with the whole-cell lysate of Balb/c peritoneal cells. In this case, too, we have demonstrated the interaction of early infection-induced antibody clones obtained from GF and SPF mice with protein targets of whole-cell lysate of peritoneal cells. The responses of both GF and SPF mice appear to be similar in intensity, but they respond differently in terms of their protein targets (Supplementary Fig. S5).

## Discussion

The immune response to natural infection with *F. tularensis* as well as the response to immunization of experimental animals or vaccination of humans with attenuated strains induced strong cell-mediated immunity. Originally, the delayed-type skin test or the in vitro lymphocyte stimulation test demonstrated the expression of cell-mediated immune response at the end of the first week after natural infection or vaccination^20,54^. Similarly, the utilization of whole-blood culture DNA synthesis response and IL-2 and IFN-gamma secretion authenticated the second week as marking the time interval needed for the Th1 type of T-cell-mediated immunity expression after vaccination of human volunteers with a live vaccine strain^55^. In parallel, it was demonstrated that a substantial antibody response of human patients occurred within the second week of infection^54^. Studies on the host genetic background of resistance to experimental *Francisella* infections^56–58^ and the participation of neutrophils, NK cells, or B cells in the resistance to primary tularemia infection^13,33,59,60^ drew attention to early innate immunity. This innate (natural, genetic, or inherent) immunity (or resistance) has been defined as “the ability of an organism to resist the effect of an ecological or physiological agent in the absence of any acquired (reactive) immunity”^61^. In other words, innate immune responses occur because cells are able rapidly to react to invading microorganisms without the requirement for an antigen-specific adaptation. Innate immune responses in the context of *Francisella* models operate until the fifth day post infection^6^. During this time period, the unspecific early protective immunity can be demonstrated in murine models between the second and third days post infection with vaccine strain of *F. tularensis*^33,62^; human volunteers respond to LVS vaccination by activation of γδ T cells and NK cells 24 h post vaccination^63^. Transcriptome analysis has demonstrated the induction of proinflammatory and innate immunity-related genes at early post-vaccination time points, meaning ≤ 48 h^64^.

Although we have been investigating immunity against *F. tularensis* infection for many decades our knowledge as to the role of natural humoral mechanisms remains relatively rudimentary. We have identified, rather serendipitously, several antibody clones isolated 12 h post infection from sera of SPF mice immunized with LVS that reacted with *F. tularensis* proteins and were absent in the sera of control mice. Such an early antibody response could be either a response of memory B cells resulting from previous interactions with microorganisms or an induction of T-cell-independent early natural antibody production induced by *F. tularensis* strain FSC200 infection. We decided to resolve this dilemma using GF mice that should not have had any interaction with bacteria during their ontogeny, and not even with bacteria that constitute the gut microbiome of SPF mice.

According to earlier studies, the antibody production of GF mice in response to corpuscular antigens, such as from sheep red blood cells^65,66^ or hamster erythrocytes^67^, was comparable to that seen in conventional mice. Similarly, only limited differences were observed between conventional and GF mice in response to hydrophilic polysaccharide dinitrophenyl-lysine-Ficoll or dinitrophenylated bovine serum albumin^66^. In all these early studies, the humoral immune response or competence was monitored in the intervals corresponding to induction of acquired immune responses. Antibody production during early time intervals corresponding to induction of innate immune responses was not in focus, despite the fact that the natural antibodies have been shown—beyond immediate protection against infection—to play a substantial regulatory role in the actions of the defense system and homeostasis^68^.

Our study revealed the early humoral innate immune response of GF as well as SPF mice to the intracellular bacterium *F. tularensis* at least from the 12th hour after intraperitoneal inoculation with the bacteria. The majority of antibody clones reacted with bacterial proteins that have orthologs or analogs in eukaryotic cells. The dominant targets were cytosolic and mitochondrial proteins. Some of the clones reacted also directly with eukaryotic proteins separated by SDS PAGE of naïve murine peritoneal cells lysate. Considering that natural antibodies (autoantibodies) have been shown to recognize evolutionarily fixed epitopes shared between foreign and self-antigens^69^, the results of our study correspond to the generally accepted target profile of natural antibodies. The isotypes composition and their relative proportions in the serum of GF mice also correspond to the general characteristics of natural antibodies. The kinetics of antibody production suggest a temporary nature of humoral innate response, at least in the sense that some antibody clones (not the B cell clones) disappear within the next 12 h. Such kinetics suggest that different (sub)types of B cells and plasma cells might participate in the early natural humoral immune response to infection. Part of the clones persists, however, and these specificities can be identified even in the acquired immune response phase. Antibody specificities that were detected in both the innate immunity phase and the acquired immunity phase may result from the B cell responses regulation, selection of the B cell repertoires, and/or the regulation of B cell development, all of which are processes modulated by early produced natural antibodies^70^. An anamnestic response of memory B cells derived from an innate B cell pool by infection may ensure selected specific and long-term acquired humoral responses.

This comparison of the GF and SPF mice antibody response intensities leads to the conclusion that previous interaction with bacteria instructed the immune system of SPF mice and that the uniform humoral response of the naïve innate immune system of GF mice is more limited, and especially at IgM isotype level. Moreover, data in the literature proves that the exogenous antigenic stimulation has changed the relative proportion of cells secreting IgM isotype antibody on the one hand and cells secreting IgG and IgA antibody isotypes on the other. This means that exogenous antigenic stimulation has a great effect on the background IgM-, IgG-, and IgA-secreting cells, and, most likely, it also changes the repertoire of antigenic specificities of the antibodies produced^71^.

It has been demonstrated that antibodies—including natural antibodies—can influence the immune processes through diverse mechanisms, among these being opsonization of microbes, complement activation, formation of circulating antigen–antibody complexes and their trapping in lymphoid organs, antibody-dependent cell-mediated bactericidal activity, signaling through Fc receptors, and modulation of the inflammatory response. Moreover, natural antibodies may prevent pathogen dissemination to target organs and influence the immunogenicity through enhanced antigen-trapping in secondary lymphoid organs^72,73^. All the activities of natural antibodies mentioned above can modulate the induction or regulation of acquired immune responses^73^.

Among the early induced natural antibody clones were clones reacting with ClpB, SodB, KatG, Hfq, IglA, IglB, and IglC proteins, which already had been demonstrated to be *Francisella* virulence factors responsible for intracellular survival and stress response^74^. In addition to the KatG and SodB proteins that are secreted, antibody clones reacting with the other secreted proteins DnaK and GroES also have been identified. It is noteworthy that antibody clones reacting with these virulence factors encompassing both unique bacterial proteins and proteins having eukaryotic orthologs or analogs, with the exception of SodB, were identified only at time point 12 h postinfection. All these antibody specificities may potentially modulate the early stages of interaction between *Francisella* and the host cell. In this context, we would mention that KL Elkins et al. have disclosed in several studies an early T-cell-independent and B-cell-dependent specific or eventually nonspecific protective response that can be demonstrated between the second and third day after primary sublethal infection of mice^33,62,75,76^. Due to the fact that, according to a notion prevailing since the late 1990s, the antibodies are produced up to about the fifth or seventh day after vaccination or infection, early resistance to *Francisella* and/or *Listeria* has not been attributed to the involvement of antibodies. According to the results presented here, however, the early production of infection-induced natural antibody clones reacting with *F. tularensis* proteins may be the critical partner of the early cellular responses controlling the presence of bacteria in the body. A search for direct proof of antibody involvement in the induction of this early anti-infective resistance should be a matter for further experiments.

## Conclusion and perspectives

We conclude that both GF and SF mice respond to *F. tularensis* strain FSC200 intraperitoneal infection by early IgM, IgG3, and IgA antibody production that can be detected in serum as early as 12 h after infection. Some antibody specificities production has a temporary character. The spectrum of 87 antibody specificities, consisting of a group of bacterial proteins, of which 63 have orthologs or analogs in eukaryotic cell proteins, along with the kinetics of their production, lead to the conclusion that this is a production of natural antibodies, probably by B-1a cells induced by *F. tularensis* intraperitoneal infection. Preliminary testing of cell subtypes infection in murine peritoneum proved B-1a cells to be the dominant target of *Francisella* invasion (data not shown). It had already been demonstrated that body cavity B-1 cells are activated by infection and can be considered—unlike lymph tissues B-1 cells—as responder cells^70,77,78^. Our results, however, do not correspond to those obtained in a model of influenza infection where no specific IgM antibodies in serum were detected at early intervals after intranasal infection^77,78^. The reason for this difference can be seen in the different cell types, and including B cell subtypes, that encounter infectious agents as first responders on the nasal mucosa and in the lung in comparison with those which are first responders in peritoneum. Differences in pathogen type (i.e., virus versus bacterium) also can play a substantial role.

Such an early production of antibodies raised several questions. First, where is the origin of this antibody production? Is it directly in the peritoneum or does it occur after translocation of B cells to lymph organs? Further, which B cell subset(s) really produce(s) these antibodies and which receptor(s) and signaling pathways are involved in this phenomenon? What is the primary target of the antibodies thus produced? Are they primarily autoantibodies or are they already targeted, due to the phylogenetic relationship between eukaryotes and prokaryotes, as well as phylogenetic memory, to components of an invading microorganism? Finally, how do these early produced antibodies participate in the *Francisella*–host cell interactions and to what extent can they influence the induction of protective immune response? The positive effects of B-1a cells and of antibodies produced early by B-1a cells already have been reported^79–81^.

The phylogenetically stabilized defense mechanism can utilize these early infection-induced antibodies both to recognize the components of the invading pathogens and to eliminate the pathogen-damaged own cells. Such early defense steps can thus be characterized as a booster of opsonophagocytosis (or neutralization) of pathogens that is one of the key events in the induction of acquired immunity mechanisms that already are targeted upon eliminating the pathogen^82^ (Table 1). Nevertheless, there remain substantial gaps in our understanding of cooperation between the early humoral and cellular branches of the innate and acquired immune systems in expression of protective responses to *F. tularensis* infection that can lead us to the design of an effective and safe vaccine.

**Table 1:** Possible phases of infection-induced natural antibodies involvement in the restoration of homeostasis after infection.

	Effector	Target	Process
Stage 1	nIg sAb	Microbial surface targets	Opsonization
Stage 2	Complement	nIg-antigen complex	Complement activation
Stage 3	C-Ig-Ag complex	CRs, IgRs and/or BCR	Cell-microbe interaction
Stage 4			B cell subset(s) activation
Stage 5			Production of i-nAb
Stage 6	i-nAb	Components of pathogen(s) and/or self infection-damaged cells	Reinforced opsonization
Stage 7	i-nAb-(C)-Ag complex	Receptors of phagocytic and/or immunocompetent cells	Elimination and destruction of pathogens and damaged self cells by phagocytes
Stage 8			Induction of adaptive immunity and immunological memory
Stage 9			Restored homeostasis

*nIg sAb* natural IgM soluble antibodies, *IgRs* immunoglobulin receptors, *i-nAb* infection induced early (natural) antibodies.

The suggested scenario could be supported by the data from *Francisella*-host cell interaction demonstrating that IgM is essential for *F. tularensis* opsonization by complement components.

## Material and methods

### Bacteria

*Francisella tularensis* subsp. *holarctica* strain FSC200 (*F. tularensis* FSC200) was kindly provided by Åke Forsberg (Swedish Defence Research Agency, Umeå, Sweden). *Klebsiella pneumonia* was a clinical isolate, and *Pseudomonas aeruginosa* CCM 3955 was obtained from Czech Collection of Microorganisms (Masaryk University, Brno, Czech Republic). Stocks of *F. tularensis* were precultivated on McLeod agar supplemented with bovine hemoglobin and IsoVitalex (Becton Dickinson, New Jersey, USA) at 37 °C for 24 h. Chamberlain medium^83^ was prepared according to the manufacturer’s instructions, pH was adjusted with HCl to 6.8 (unless otherwise stated), and the medium was sterile-filtered rather than autoclaved. Following incubation, bacterial colonies were lifted from the plates and resuspended in saline to reach optical density 1.00 (corresponding to bacterial 5 × 10^9^ CFU/ml). The actual number of bacteria in the suspension utilized for the experiments was determined by serial dilutions and the number of colony-forming units (CFU) was calculated. *Klebsiella pneumonia* and *Pseudomonas aeruginosa* CCM 3955 were cultivated under standard cultivation procedures using blood agar plates.

### Animals

Female BALB/c-SPF mice (SPF) were purchased from Velaz (Unetice, Czech Republic). Female germ-free (GF) BALB/c (hereafter GF mice) mice were kindly provided by the Institute of Microbiology of the Czech Academy of Sciences (Department of Gnotobiology, Novy Hradek, Czech Republic). The GF mice were housed under sterile conditions in Trexler-type plastic isolators with free access to autoclaved tap water and 50 kGy-irradiated sterile pellet diet Altromin 1414 (Altromin, Lage, Germany). Before experiments GF mice were transferred into sterile indiviually ventilated cages (IVC, Tecniplast, Italy). The SPF mice were housed under specific-pathogen-free conditions and GF mice were housed in micro-isolator cages under germ-free conditions. The mice were placed into sterile boxes with air-conditioning and stabilized temperature of 22 ± 2 °C. Light mode was 12 h of light and 12 h darkness^45^. All experiments on mice were conducted under supervision of the institution’s Animal Unit and were approved by the Animal Care and Use Committee of the Faculty of Military Health Sciences, University of Defence, Hradec Kralove, Czech Republic under project number 35/17**.** All experiments were carried out in compliance with the ARRIVE guidelines and regulations (http://www.nc3rs.org.uk/page.asp?id=1357).

### Preparation of bacterial whole-cell lysates

The suspensions of bacterial cells (*F. tularensis*, *Klebsiella pneumonia*, *Pseudomonas aeruginosa*) were disrupted using a French press twice at 16,000 psi and cell debris was removed by centrifugation at 12,600×*g* for 30 min at 4 °C. Benzonase nuclease (250 U/μL, Sigma, St. Louis, MO, USA) was added to the supernatant, resulting in a final concentration of 0.5 U/ml of lysate.

### Preparation of eukaryotic whole-cell lysate

For peritoneal cavity lavage, the mice were anesthetized under complete anesthesia; the abdominal skin was cleaned thoroughly with ethanol pads. For each mouse, a sterile tweezer was used to pinch and lift the middle section of the abdominal skin approximately 2 cm to create a pocket of peritoneum void of internal organs. A syringe with needle was filled with 10 ml phosphate-buffered saline (PBS) and the needle was inserted vertically into the pocket within the peritoneal cavity while being cautious to avoid injuring the internal organs. The PBS solution was injected into the peritoneum. Recovery of the lavage fluid was in the range of about 80–90%, depending on the peritoneal fat content and lavage technique. The retrieved suspension was centrifuged at 400×*g* for 5 min to concentrate any cellular components. Peritoneal lavage cells were then lysed with RIPA buffer (Thermo Fisher Scientific, USA) to obtain extracted proteins. The extraction was done according to the manufacture’s procedure. The protein suspension was subsequently precipitated using trichloroacetic acid/sodium deoxycholate (TCA-DOC) with acetone wash. Briefly, the protein suspension was mixed with 1/10 of the volume of 0.15% sodium deoxycholate (DOC) and incubated on ice for 15 min. The sample was then mixed with 1/10 of the original volume of 72% trichloroacetic acid (TCA) and the suspension was again incubated on ice for 15 min. Subsequently, the mixture was microfuged at 12,000×*g*, at 4 °C for 15 min. Supernatant was discarded and 1 ml of acetone chilled to − 20 °C was added. All steps of TCA-DOC precipitation were repeated twice. The pellet was air-dried for 5 min, subsequently resuspended in a minimal volume with 2-D sample buffer, then subjected to 2-D gel analysis.

### Infection of mice and collection of murine sera

The SPF and GF mice were intraperitoneally inoculated with an *F. tularensis* FSC200 dose of 10^2^ CFU in 0.2 ml total volume of saline. At three time intervals post-challenge (12 h, 24 h, and 48 h), infected mice were killed by cervical dislocation, blood was collected, and serum was prepared according to a common procedure. Sterility of serum was determined by plating on McLeod agar plates for 72 h. Obtained serum was pooled and tested for specific antibody titers using enzyme-linked immunosorbent assay (ELISA). Serum aliquots were stored in volume 500 μl sera at − 80 °C. Normal murine serum was prepared from blood of Balb/c mice without exposure to any *F. tularensis* strain.

### Serum analysis procedures

#### Determination of antibody isotypes

The immunoglobulin subclasses of serum samples were determined using a Quantibody mouse immunoglobulin isotype array (RayBiotech Life, Peachtree Corners, GA, USA) while following the manufacturer’s instructions. The mouse array is a glass chip-based multiplex assay that detects the 8 mouse immunoglobulin subclasses (IgG1, IgG2a, IgG2b, IgG3, IgA, IgD, IgE, and IgM) plus the 2 light chain types (kappa and lambda). Briefly, samples were mixed with the Cy3 equivalent dye-labeled detection antibody and applied to the array slide. After a wash step, the slides were dried and scanned with a laser scanner. Results were evaluated by densitometric data extraction (Quantibody Q Analyzer, RayBiotech Life, Peachtree Corners, GA, USA) (https://www.raybiotech.com/analysis-tools/).

#### ELISA-based method for titer determination

ELISA laboratory technique was selected to assess the magnitude of humoral immune response for evaluating the time course and immune status of an ongoing infection. Briefly, ELISA plates were coated with *F. tularensis* FSC200 autolysate Ag-2 in concentration corresponding to 1.1 × 10^9^ bacteria/well and incubated overnight at 4 °C. The following day, plates were washed with PBS/Tween 20 (0.05% [v/v]) solution and blocked with 200 μl of 10% calf serum in PBS for 30 min at 37 °C. After washing, serum samples were serially diluted with PBS onto plates and incubated at 37 °C for 90 min. Subsequently, plates were washed and enzyme-labeled antibodies were added for another 90 min at 37 °C. Polyclonal HRP-conjugated goat anti-mouse Ig (Bio-Rad AbD Serotec Limited, Oxford, UK) was used for detection. Subsequently, plates were washed and TMB solution was used for color development. Final optical density values were measured at 450 nm using a plate reader MultiDetector Magic XBC Paradigm Detection Platform (Beckman Coulter, Pasadena, CA, USA). Uncoated plates were also assayed to ensure that antibodies in immune sera did not bind a plastic. Antibody titers were expressed as optical density values obtained after subtraction of the blank value, which was obtained from the duplicate wells containing wash buffer.

### Immunoproteomic analysis of murine sera

#### Mini two-dimensional electrophoresis

Bacterial whole-cell lysates were solubilized in isoelectric focusing (IEF) rehydration buffer containing 6 M urea, 2 M thiourea, 4% CHAPS, 40 mM TRIS-base, 0.12% De Streak, and 0.5% bromophenol blue with 1% carrier ampholytes (pH 3–10) and 0.5% carrier ampholytes (pH 6–11). Protein concentration was defined by bicinchoninic acid assay. The IEF was performed using a Multiphor II electrophoresis system (GE Healthcare, Chicago, Illinois, USA) on 7 cm gradient pI 3–10 Immobiline DryStrip gels^84^. Briefly, proteins were loaded by in-gel rehydration onto polyacrylamide gel strips with a nonlinear immobilized 3–10 pH gradient (IPG) from GE Healthcare (Chicago, IL, USA) and separated according to their different pI values by IEF. Following IEF, the IPG strips were treated in equilibration buffer containing 2% (w/v) sodium dodecyl sulfate (SDS), 50 mM Tris/HCl (pH 8.8), 6 M urea, 30% (v/v) glycerol, and 1% (w/v) dithiothreitol (DTT). This was immediately followed by a second equilibration of strip in the same solution containing 4% (w/v) iodoacetamide (instead of DTT). In the second dimension, the IPG strips were embedded onto 12% homogeneous SDS polyacrylamide gels. Electrophoresis was performed on a Protean II Multi-Cell (Bio-Rad, Hercules, CA, USA) according to the standard running conditions^84^. Blue staining was then used to visualize proteins on a reference protein map while colloidal blue staining was performed for mass-spectrometric purposes. Analyses of reference maps were made by visual inspection and data were evaluated using Melanie 3 software (Bio-Rad Laboratories, Hercules, CA, USA)^84^.

#### Semi-dry western blotting

Selected 2-DE gels were electroblotted onto polyvinylidene difluoride membrane. All immunoreactive spots were detected using incubation with control or immune sera diluted 1:100 in the blocking buffer at 4 °C overnight. The next day, membranes were washed with 0.05% Tween 20 in Tris-buffered saline. Polyclonal peroxidase-conjugated goat anti-mouse IgG antibody (Dako, Copenhagen, Denmark) or monoclonal HRP-conjugated goat anti-mouse IgM antibody (Invitrogen, Carlsbad, Ca, USA) was diluted 1:100 in blocking buffer and used for secondary antibody detection. Membranes were then washed and prepared for visualizing using BM Chemiluminescence Blotting Substrate kit (Boehring, Mannheim, Germany) and CL-XPosure films (Pierce, Rockford, IL, USA).

#### Mass spectrometry profiling

##### In-gel digestion

Selected protein spots were excised from representative colloidal blue-stained gels and subjected to in-gel digestion. Briefly, gel pieces were covered with 100 mM Tris/HCl (pH 8.5) in 50% acetonitrile (ACN) at 30 °C for 20 min. Next, 50 mM ammonium bicarbonate (pH 7.8) in 5% ACN was added to each gel piece. Subsequently, gel pieces were vacuum-dried, covered with 0.1 μg of trypsin in 50 mM ammonium bicarbonate (pH 7.8) in 5% ACN, then gently shaken at 37 °C overnight. Protein digests were then extracted using 2% trifluoroacetic acid and 98% ACN. The final extracts were reduced using a vacuum dryer to approximately 15 μl and frozen at − 80 °C. The resulting peptides were mixed with 5 mg/ml of α-cyano-4-hydroxycinnamic acid in 50% acetonitrile and 0.1% trifluoroacetic acid. Peptide samples with matrix solution were spotted onto a MALDI plate.

##### Protein identification and characterization

The protein identification and characterization analysis of the murine sera have been done according^45,84^. Briefly, the mass spectra were recorded in the positive reflectron mode on a 4800 MALDI-TOF/TOF mass spectrometer (AB Sciex, Foster City, CA, USA) and operated in its delayed extraction mode. Internal calibration of mass spectra was conducted utilizing the tryptic autolytic peptides. Acquired data were evaluated using GPS Explorer Software v.3.6 (AB Sciex, Australia) (https://sciex.com/products/software), which integrates the Mascot search algorithm against the *Francisella tularensis* subsp. *holarctica* OSU18 genome database. The following parameters were used for protein identification: 100 ppm error, optional oxidation of methionine, fixed carbamidomethylation of cysteine residues, and one possible missed cleavage site allowed. Proteins were considered to be identified with confidence when a minimum of two peptide sequences per protein were identified and the GPS protein score confidence interval was equal to 100%. Trypsin was selected as the proteolytic enzyme. The identities of all proteins were determined according to the UniProtKB database^85^ (https://www.uniprot.org).

#### Bioinformatic analysis

A number of online programs were used for bioinformatic analysis and characterization of identified proteins. EggNOG 5.0.0 algorithm was applied to sort the *Francisella*-identified proteins into functional categories^86^ (http://eggnog5.embl.de/#/app/results). PSORTb 3.0. was used to predict localization based on the ORF sequences of identified proteins^87^ (https://www.psort.org/psortb/). SignalP 5.0 was used for predicting signal peptide presence (type II secretion pathway)^88^ (http://www.cbs.dtu.dk/services/SignalP/). LipoP 1.0 was applied to predict lipoproteins and to discriminate between lipoprotein signal peptides, other signal peptides, and n-terminal membrane helices in Gram-negative bacteria^89^ (http://www.cbs.dtu.dk/services/LipoP/).

### Statistical analysis

Each immunoblot experiment was conducted at least in triplicate while using three biological replicates. Pearson correlation coefficient was calculated using GraphPad Prism 5.01 software (GraphPad Software, San Diego, CA, USA) (https://www.graphpad.com/scientific-software/prism/). All statistical analyses were also performed using GraphPad Prism 5.01 software (GraphPad Software, San Diego, CA, USA). In case of Quantibody immunoglobulin isotype array, four technical replicates were measured (according to the manufacturer’s instructions). Reference antibody isotypes and light kappa and lambda chains are presented as well as reference immunoblots. Values are expressed as mean ± standard deviation (SD) and analyzed for significance using Student’s two-tailed *t*-test. Differences were considered statistically significant at **p < 0.01 and *p < 0.05, unless otherwise stated, and are denoted by asterisks.

## Supplementary Information

Supplementary Information

## Data Availability

All data generated or analysed during this study are included in this published article (and its Supplementary Information files).
