# Supplementary material for: Early infection-induced natural antibody response
Source: Sci Rep. 2021 Jan 15;11:1541. doi: 10.1038/s41598-021-81083-0 (PMC7810682; doi:10.1038/s41598-021-81083-0)
Supplement: Supplementary file 1 — Supplementary Information [file 41598_2021_81083_MOESM1_ESM.pdf]

Supplementary information

Early infection-induced natural antibody response

Kubelkova Klara[1*], Hudcovic Tomas[2], Kozakova Hana[2], Pejchal Jaroslav[3], and Macela Ales[1]

[1] *Department of Molecular Pathology and Biology, Faculty of Military Health Sciences, University of Defence, Trebesska 1575, 500 01 Hradec Kralove, Czech Republic*

[2] *Department of Immunology and Gnotobiology of the Institute of Microbiology of the Academy of Sciences of the Czech Republic, Videnska 1083, 142 20 Prague 4, Czech Republic*

[3] *Department of Toxicology and Military Pharmacy, Faculty of Military Health Sciences, University of Defence, Trebesska 1575, 500 01 Hradec Kralove, Czech Republic*

* Correspondence:

Klara Kubelkova, Ph.D.
Department of Molecular Pathology and Biology
Faculty of Military Health Sciences
University of Defence
Trebesska 1575
500 01 Hradec Kralove
Czech Republic

Email: klara.kubelkova@unob.cz
Tel: +420 973 255 193

Supplementary Figure S1……………………………………………………………………3

Supplementary Figure S2……………………………………………………………………4

Supplementary Figure S3……………………………………………………………………5

Supplementary Figure S4……………………………………………………………………6

Supplementary Figure S5……………………………………………………………………7

Supplementary Table S1……………………………………………………………………8

Supplementary Table S2……………………………………………………………………15

Supplementary Figure S1

Isotypes of control Balb/c GF mice sera and Balb/c SPF mice sera without any targeted bacterial colonization. The basal individual antibody isotypes levels for GF mice were significantly lower in comparison with the corresponding isotypes levels for SPF mice. Quantibody mouse immunoglobulin isotype arrays were performed in biological triplicate (individual sera) for all time intervals and were independently repeated at least three times. The reference isotypes are presented. Values are expressed as mean ± standard deviation (SD) and analyzed for significance using Student's two-tailed *t*-test.

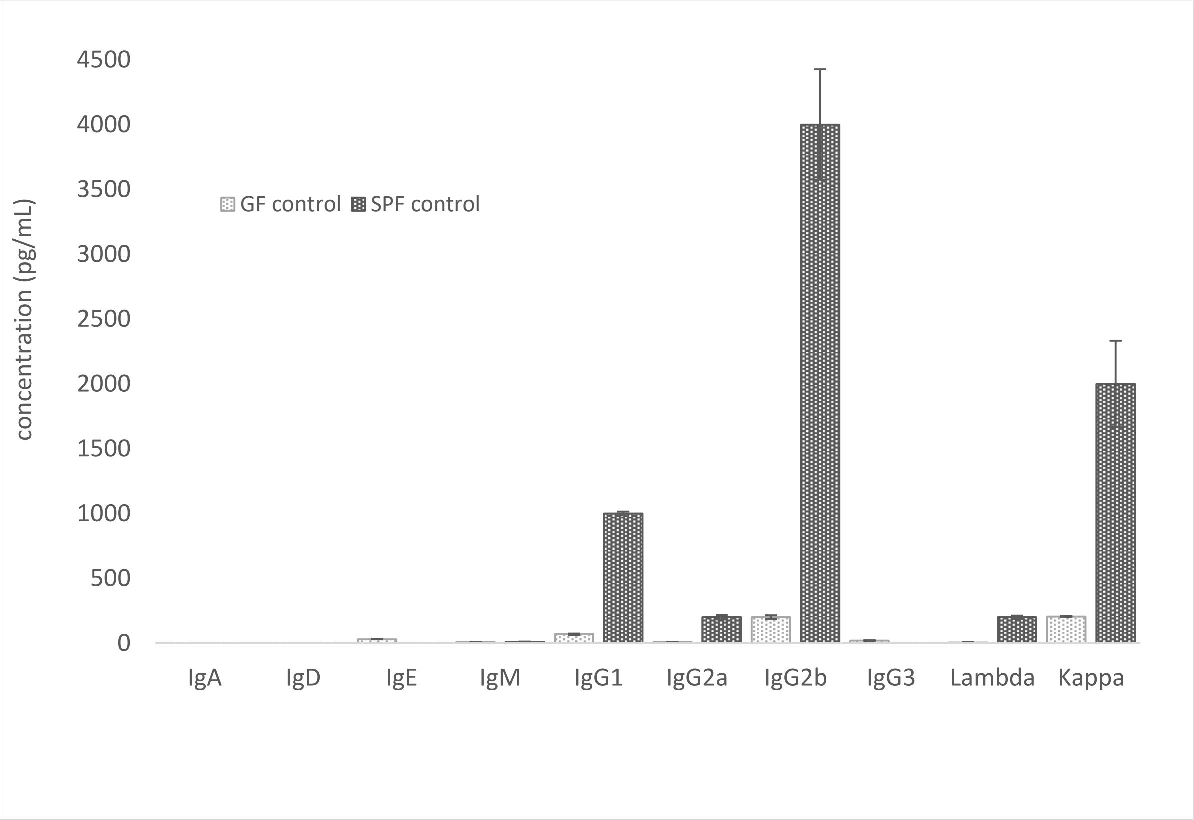

Supplementary Figure S2

The levels of light kappa and lambda chains in analyzed Balb/c GF mice sera and Balb/c SPF mice sera. Quantibody mouse immunoglobulin isotype arrays were performed in biological triplicates (individual sera) for all time intervals and were independently repeated at least three times. The reference levels of light kappa and lambda chains are presented. Values are expressed as mean ± standard deviation (SD) and analyzed for significance using Student's two-tailed *t*-test.

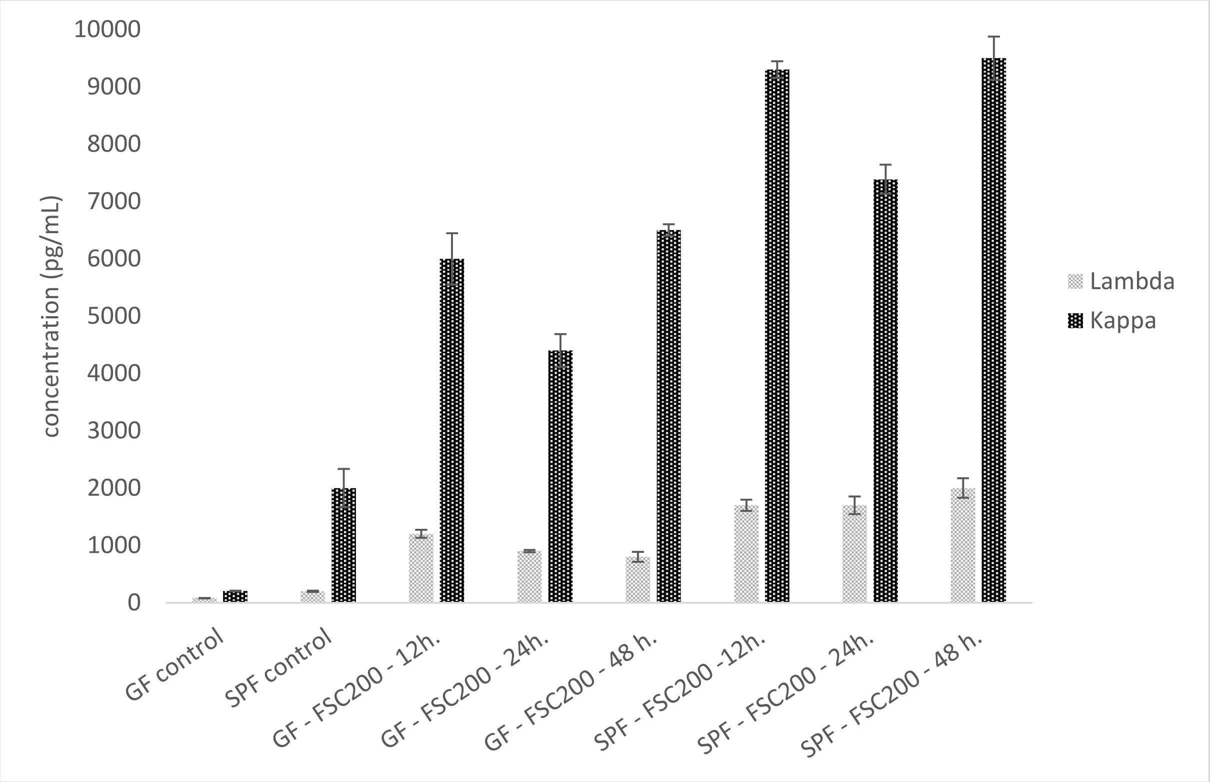

Supplementary Figure S3

Reference 2-D immunoblots of *F. tularensis* FSC200 whole-cell lysate protein targets, which are recognized by mice sera obtained from *F. tularensis* FSC200 infected Balb/c mice 12 h post infection using anti IgM as a secondary antibody. IgM antibody clones of Balb/c GF mice are visualized in upper blot, IgM antibody response of SPF mice is demonstrated on bottom blot. A total 200 µg of protein was separated by immobilized pH gradient (IPG) strips (3–10) and 12% (w/v) SDS-PAGE gels. Monoclonal HRP-conjugated goat anti-mouse IgM antibody was used for secondary antibody detection by chemiluminescence. No spots were excised for further MS/MS analysis (not applicable). All experiments were performed in biological triplicates (individual sera) for all time intervals and were independently repeated at least three times.

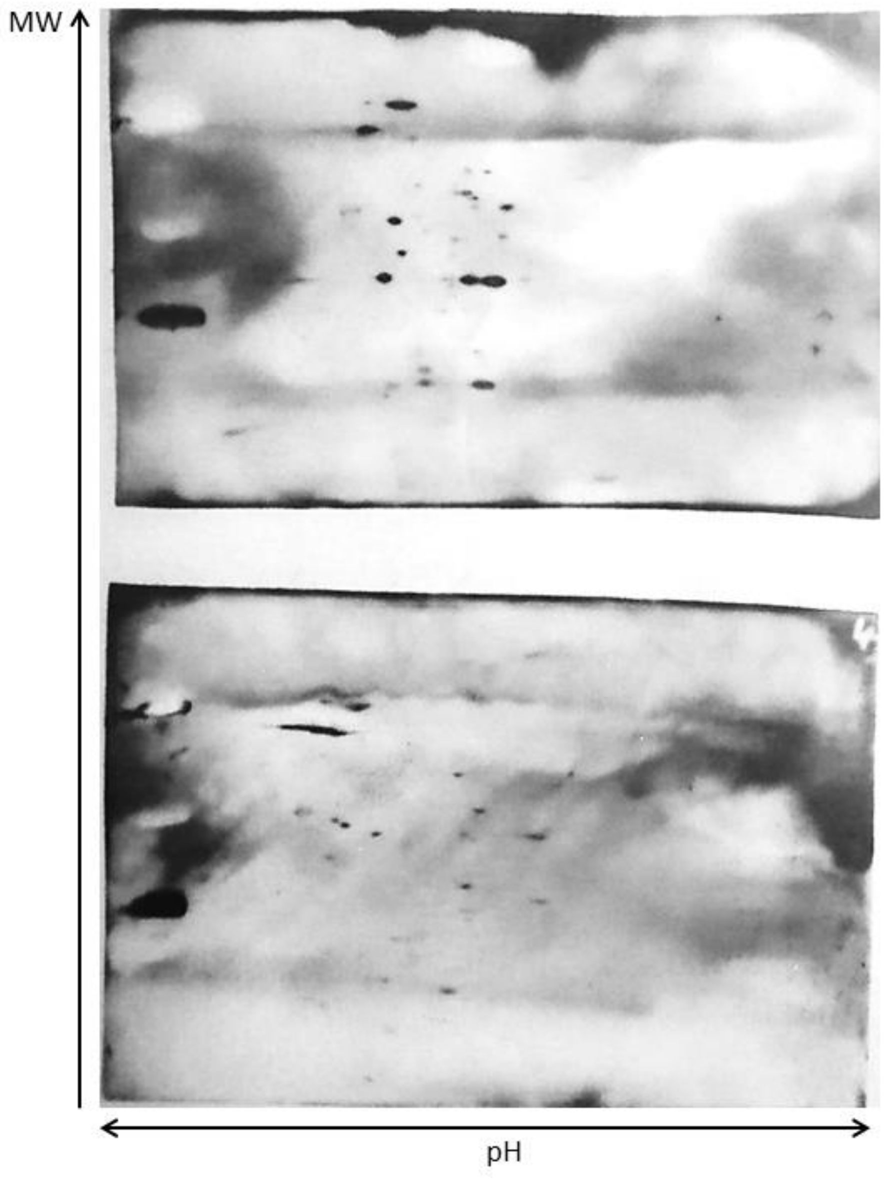

Supplementary Figure S4

Reference 2-D immunoblots of Balb/c mice whole-cell lysate of **(a)** *Klebsiella pneumoniae* and **(b)** *Pseudomonas aeruginosa*, which are recognized by mice sera from *F. tularensis* FSC200 vaccinated Balb/c mice. The sera with antibody clones reacted with host cell proteins were obtained from Balb/c GF mice (upper blots) and SPF mice (bottom blots) 12 h after infection. A Total 200 μg of protein was separated by immobilized pH gradient (IPG) strips (3–10) and 12% (w/v) SDS-PAGE gels. Polyclonal peroxidase-conjugated goat anti-mouse IgG antibody was used for secondary antibody detection by chemiluminescence. No spots were excised for further MS/MS analysis (not applicable). All experiments were performed in biological triplicates (individual sera) for all time intervals and were independently repeated at least three times.

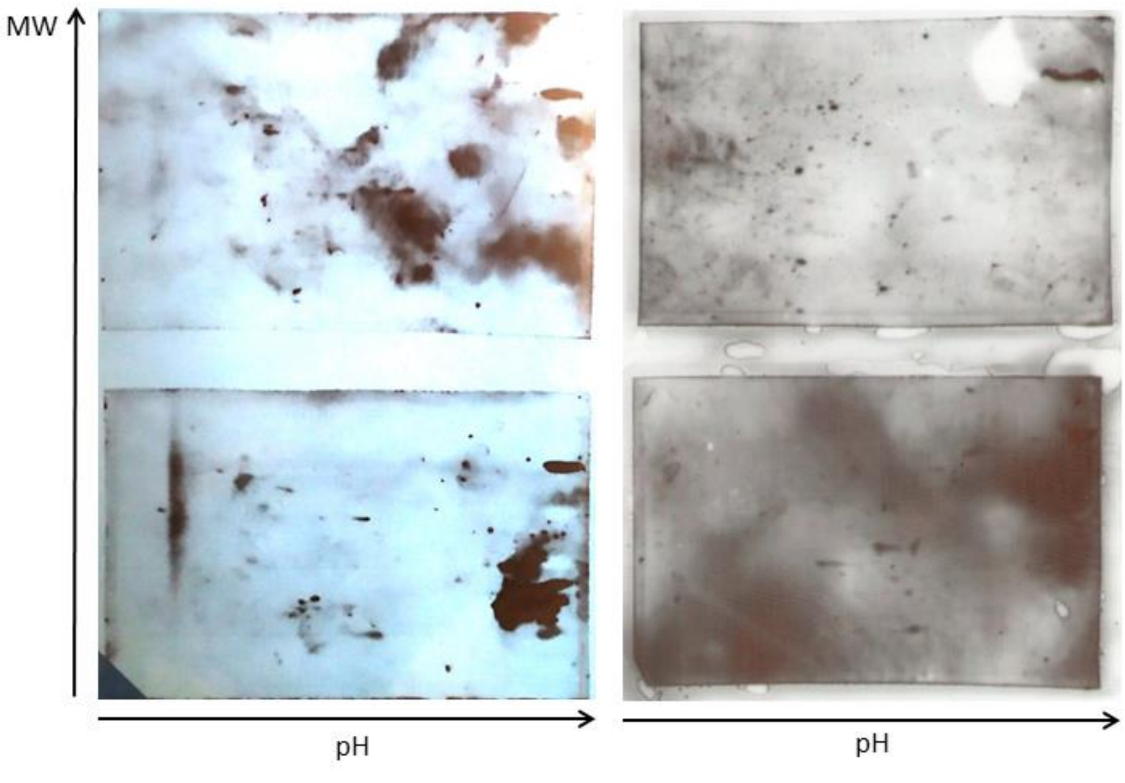

Supplementary Figure S5

Reference 2-D immunoblots of Balb/c mice whole-cell lysate of peritoneal lavage cells, which are recognized by mice sera from *F. tularensis* FSC200 vaccinated Balb/c mice. The sera with antibody clones reacted with host cell proteins were obtained from Balb/c GF mice (upper blot) and SPF mice (bottom blot) 12 h after infection. A total 200 µg of protein was separated by immobilized pH gradient (IPG) strips (3–10) and 12% (w/v) SDS-PAGE gels. Polyclonal peroxidase-conjugated goat anti-mouse IgG antibody was used for secondary antibody detection by chemiluminescence. No spots were excised for further MS/MS analysis (not applicable). All experiments were performed in biological triplicates (individual sera) for all time intervals and were independently repeated at least three times.

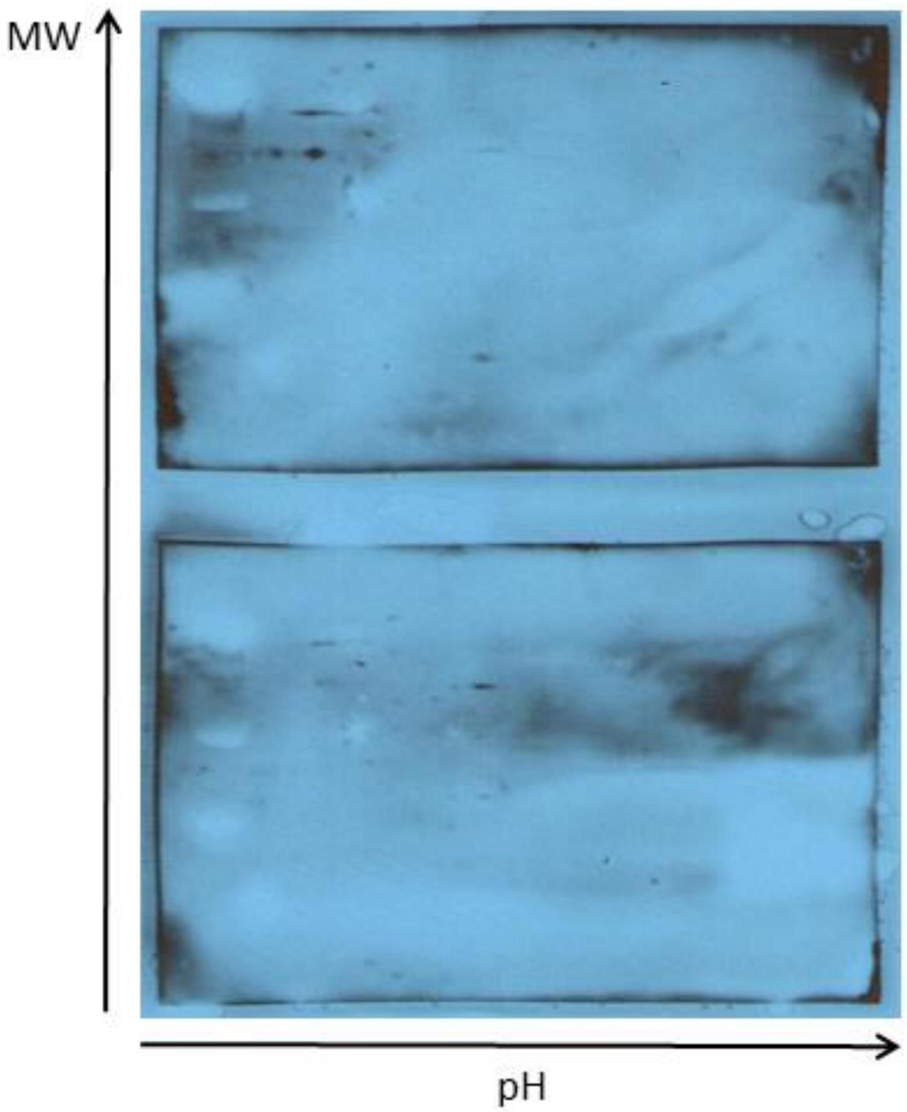

Supplementary Table S1

Spot No.	Gene locus	Name of protein	Immunoreactivity of tested sera						Acc. No.[a]	Gene	Mr/pI (theor.)[b]	Mr/pI (meas.)	SignalP[c]	PSORTb[d]	LipoP[e]	Ref.
			GF 12h.	GF 24h.	GF 48h.	SPF 12h.	SPF 24h.	SPF 48h.								
1B,2B 1D	**FTS_1167**	Chaperone protein DnaK	-	+	-	-	+	-	**AFT92942**	dnaK	69.2/4.88	69.3/4.88	No	cyt	cyt	[1,2,4-6,6a,7,9-12]
2D	**FTS_0019**	Aspartate-tRNA synthetase	-	-	-	-	+	-	**AFT92064**	aspS	67.0/5.43	66.9/5.49	No	cyt	cyt	
3	**FTS_0084**	Chaperone ClpB	+	-	-	-	-	-	**AFT92111**	clpB	96.0/5.38	96.1/ 5.38	No	cyt	cyt	[4,6,6a,8,12]
3D	**FTS_1893**	Putative ABC transporter ATP-binding protein	-	-	-	-	+	-	**AFT93475**	-	63.0/5.26	63.1/5.26	No	cyt	cyt	[3,5,12]
4,5,6 8	**FTS_1471**	Peroxidase/Catalase	+	-	-	+	+	-	**AFT93160**	katG	82.6/5.43	82.5/5.43	Yes	Un	SpI	[3-6,6a,8, 10-12]
3C,4C 5C	**FTS_1670**	Chaperonin GroEL	-	-	+	-	-	-	**AFT93305**	groEL	57.4/4.99	57.5/4.99	No	cyt	cyt	[2,4-12]
6B,7B	**FTS_1126**	Intracellular growth locus protein B	-	+	-	-	-	-	**AFT92909**	iglB2	58.0/4.76	59.2/4.74	No	cyt	cyt	[6-8,11]
6D	**FTS_1877**	Bifunctional Phosporibosyl-aminoimidazolecarboxamide formyltransferase/IMP cyclo-hydrolase	-	-	-	-	+	-	**AFT93463**	purH	56.3/5.88	56.8/5.88	No	cyt	cyt	
7	**FTS_0527**	Fumerate hydratase	+	-	-	-	-	-	**AFT92445**	fumA	55.1/5.27	55.3/5.27	No	cyt	cyt	
7D	**FTS_0877**	(Dimethylallyl)adenosine tRNA methylthiotransferase	-	-	-	-	+	+	**AFT92720**	miaB	50.1/5.86	50.5/5.85	No	cyt	cyt	
8B	**FTS_1797**/	Aspartyl/glutamyl-tRNAamidotransferase subunit A	-	+	-	-	-	-	**AFT93411**	gatA	52.4/5.41	52.7/5.41	No	cyt	cyt	
8D,9D 10D,13 15	**FTS_1709**	Elongation factor Tu	+	+	+	-	+	+	**AFT93339**	tufA	43.6/5.12	43.4/5.11	No	cyt	cyt	[2,4,6a,8,11, 12]
9	**FTS_1116**	Transketolase	+	-	-	-	-	-	**AFT92902**	tktA	73.3/5.85	73.6/5.85	No	cyt	cyt	[4]
9B	**FTS_0675**	3-methyl-2-oxobutanoate hydroxymethyltransferase	-	+	-	-	-	-	**AFT92559**	panB	28.9/5.55	32.1/5.58	No	Un	cyt	

10	**FTS_0882**	Trigger factor	+	-	-	+	-	-	**AFT**92724	tig	49.6/5.0	49.6/5.00	No	cyt	cyt	
11	**FTS_1457**	Phosphoglyceromutase	+	-	-	-	-	-	**AFT93147**	gpml	57.6/5.83	58.6/5.90	No	cyt	cyt	
11B	**FTS_0324**	FKBF-type peptidyl-prolyl cis-trans isomerase	-	+	+	-	-	-	**AFT92298**	fkpB	17.4/4.79	17.4/4.79	No	cyt	cyt	
11D	**FTS_1120**	Fructose-1,6-bisphosphate aldolase	-	-	-	-	+	-	**AFT92906**	fbaA	38.2/5.34	38.4/5.34	No	cyt	cyt	[10]
12	**FTS_1753**	F0F1 ATP synthase subunit alpha	+	+	+	-	-	-	**AFT93372**	aptA	55.5/4.94	55.7/4.94	No	cyt	cyt	[8]
12B	**FTS_1552**	Acetyl-CoA carboxylase, biotin carboxy carrier protein subunit	-	+	+	-	-	-	**AFT93219**	accB	16.5/5.08	16.5/5.12	No	Non-cyt (signalP)	cyt	[1,5,7,9,11, 12]
12D,71	**FTS_1486**	2-amino-3-ketobutyrate coenzyme A ligase	-	-	-	-	+	-	**AFT93169**	kbl	44.0/5.61	45.0/5.79	No	cyt	cyt	
13B	**FTS_1440**	Transcription elongation factor GreA	-	+	+	-	-	-	**AFT93131**	greA	17.7/4.79	17.7/4.79	No	cyt	cyt	
13D,75	**FTS_0609**	Transcription termination factor Rho	+	-	-	-	+	-	**AFT92508**	rho	47.1/5.77	47.2/5.77	No	CM	cyt	
14	**FTS_0088**	Tryptophan synthase subunit beta	+	-	-	-	-	-	**AFT92114**	trpB	43.3/6.9	43.1/6.90	No	cyt	cyt	
14B	**FTS_0010**	Preprotein translocase subunit SecB	-	+	-	-	-	-	**AFT92058**	secB1	16.9/4.82	17.0/4.82	No	cyt	cyt	
14D,18B	**FTS_1204**	SufS activator complex, sufC subunit	-	+	+	-	+	+	**AFT92967**	sufC	27.4/5.80	27.4/5.80	No	cyt	cyt	
15B	**FTS_0290**	Protein-export protein SecB	-	+	+	-	-	-	**AFT92274**	secB2	16.7/4.71	16.8/4.71	No	cyt	cyt	
15D,23C	**FTS_1818**	Protein-L-isoaspartate O-methyltransferase	-		-	-	+	-	**AFT93427**	pcm	23.2/5.25	23.4/5.25	No	cyt	cyt	
16,19 20,35 81	**FTS_1295**	OmpA family protein	+	+	-	-	-	-	**AFT93032**	fopA	41.9/5.58	41.9/5.58	Yes	OM	SpI, TMH	[1,5,6a,7, 10-12]
16B	**FTS_0610**	Thioredoxin	-	+	-	-	-	-	**AFT92509**	trx1	12.0/4.75	12.2/4.75	No	cyt	cyt	[2]
16D	**FTS_1406**	D-Ribulose-phosphate 3-epimerase	-	-	-	-	+	+	**AFT93112**	rpe	24.0/5.29	24.2/5.15	No	cyt	cyt	
17,26 27	**FTS_1517**	Succinyl-CoA synthetase subunit beta	+	+	+	+	+	+	**AFT93196**	sucC	41.5/5.24	41.7/5.24	No	cyt	cyt	

17B,60	**FTS_1201**	Hypothetical protein	+	+	-	-	-	-	**AFT92965**	-	25.7/7.71	25.7/7.71	Yes	Un	SpI, SPII	[11]
17D	**FTS_0889**	Host factor I for bacteriophage Q beta replication	-	-	-	-	+	+	**AFT**92731	hfq	12.5/4.87	12.5/4.93	No	cyt	cyt	[4]
18	**FTS_0935**	GTP depending nucleic acid-binding protein EngD	+	+	+	+	-	-	**AFT92764**	ychF	40.2/4.97	40.5/4.97	Yes	Un	SpI	
19A	**FTS_1432**	Purine nucleoside phosphorylase	+	-	-	-	-	-	**AFT93128**	deoD	26.9/5.87	27.1/5.87	No	Un	cyt	
19D	**FTS_0923**	Hypothetical protein	-	-	-	-	+	-	**AFT92758**	-	19.1/5.85	19.2/5.85	No	Un	cyt	
20A,64	**FTS_1518**	Succinyl-CoA synthetase subunit alpha	+	-	-	-	-	-	**AFT93197**	sucD	30.1/6.24	30.3/6.24	No	Un	cyt	
20D	**FTS_0387**	N5-carboxyaminoimidazole ribonucleotide mutase	-	-	-	-	+	-	**AFT92340**	purE	17.2/6.41	17.3/6.41	No	Un	cyt	
21A	**FTS_0013**	Single-stranded DNA-binding protein	+	-	-	-	-	-	**AFT92061**	ssb	17.5/5.63	17.5/5.63	No	cyt	cyt	[4]
22,23 24	**FTS_0258**	DNA-directed RNA polymerase subunit alpha	+	-	+	-	-	-	**AFT92248**	rpoA	35.6/4.93	35.4/4.93	No	cyt	cyt	[5,8,10]
24C	**FTS_0008**	Outer membrane protein of unoknown function	-	-	+	-	-	-	**AFT92056**	-	19.5/4.77	21.0/5.33	Yes	cyt	SpI	[7]
25	**FTS_0001**	DNA polymerase III beta subunit	+	-	-	-	-	-	**AFT92054**	dnaN	41.7/5.3	41.9/5.3	No	cyt	cyt	
27C	**FTS_0309**	Pyruvate dehydrogenase subunit E1	-	-	+	-	-	-	**AFT92287**	aceE	100.3/5.57	100.6/5.57	No	cyt	cyt	[2,6-8,10,11]
28	**FTS_1656**	Fructose-1,6-bisphosphatase II	+	-	-	-	+	-	**AFT93294**	**glpX**	34.8/5.27	35.0/5.27	No	cyt	cyt	
29	**FTS_1476**	Glycerophosphoryl diester phosphodiesterase	+	+	+	+	+	+	**AFT**93162	**ugpQ**	38.8/5.39	39.1/5.39	Yes	OM	SpI	[2,6a,10,13]
29A	**FTS_0351**	Ribonuclease PH	+	-	-	-	-	-	**AFT92313**	rph	25.4/5.03	25.5/5.03	No	cyt	cyt	
30A	**FTS_1735**	Triosephosphate isomerase	+	-	-	-	-	-	**AFT93354**	tpiA	27.7/5.04	27.9/5.07	No	cyt	cyt	[2]
30/31	**FTS_1532**	Thioredoxin reductase	+	-	-	-	-	+	**AFT93206**	trxB	34.0/4.99	34.2/4.99	No	cyt	cyt	
31/32	**FTS_1510**	Pyridoxal biosynthesis lyase PdxS	+	+	+	-	-	+	**AFT93190**	pdxS	30.8/5.18	31.0/5.18	No	cyt	cyt	
32A	**FTS_0911**	Isoprenoid biosynthesis protein	+	-	-	-	-	-	**AFT92748**	elbB	23.7/4.55	23.9/4.55	No	cyt	cyt	

33	**FTS_1166**	Heat shock protein GrpE	+	+	+	-	-	-	**AFT92941**	grpE	22.0/4.81	22.0/4.81	No	cyt	cyt	[2,4]
33A	**FTS_0787**	Adenylate kinase	+	-	-	-	-	-	**AFT92647**	adk	24.4/7.78	24.4/7.78	No	cyt	cyt	
34	**FTS_0178**	Elongation factor P	+	-	-	-	-	-	**AFT92175**	efp	20.9/4.79	20.9/4.82	No	cyt	cyt	
34A	**FTS_1862**	30S ribosomal protein S1	+	-	-	-	-	-	**AFT93455**	rpsA	61.7/5.20	63.9/5.28	No	cyt	cyt	[5,8,10,11]
36	**FTS_0836**	Isochorismatase hydrolase family protein	+	-	+	-	-	-	**AFT92684**	-	21.3/5.44	21.5/5.44	No	cyt	cyt	
37	**FTS_1359**	Guanylate kinase	+	-	+	-	-	-	**AFT93074**	gmk	21.9/4.97	22.0/5.06	No	cyt	cyt	
38	**FTS_1702**	50S ribosomal protein L7/L12	+	+	+	+	+	+	**AFT93333**	rplL	12.8/4.62	12.9/4.62	No	cyt	cyt	[1,2,4,6a, 10,11]
39,41 44	**FTS_0617**	Bacterioferritin	+	+	+	-	-	+	**AFT92513**	bfr	18.8/5.46	18.4/5.60	No	cyt	cyt	[2,3,6,6a,8]
40	**FTS_1671**	Co-chaperonin GroES	+	+	-	-	-	-	**AFT93306**	groES	10.3/5.46	10.3/5.47	No	cyt	cyt	[2,4]
42	**FTS_1002**	50S ribosomal protein L9	+	+	-	-	-	+	**AFT92815**	rpII	16.4/5.55	16.1/5.55	No	cyt	cyt	[2,8,11]
43	**FTH_0376**	Probable mannose-6-phosphate isomerase	+	-	-	-	-	+	**ABI82376**	-	14.0/5.30	14.0/5.30	No	cyt	cyt	
45	**FTS_1366**	Hypothetical protein	+	-	-	-	+	+	**AFT93081**	-	15.4/5.89	15.5/5.89	No	cyt	cyt	
46	**FTS_1795**	Hypothetical protein	+	-	-	-	-	+	**AFT93409**	-	16.2/5.93	16.2/5.94	No	cyt	cyt	
47	**FTS_1750**	ATP synthase epsilon chain	+	+	-	-	-	-	**AFT93369**	atpC	15.7/6.75	15.7/6.75	No	cyt	cyt	
48	**FTS_0815**	Hypothetical protein	+	-	-	-	-	-	**AFT92667**	-	13.1/8.33	13.2/8.33	Yes	Un	SpI, SpII	
49,52	**FTS_1155**	Sigma 54 modulation protein	+	-	-	+	-	-	**AFT92930**	yhbB	11.1/6.18	11.2/6.19	No	cyt	cyt	[2,4,10]
51	**FTS_1046**	Putative uncharacterized protein	+	-	-	-	-	-	**AFT92852**	-	12.7/6.12	12.7/6.12	No	cyt	cyt	
53	**FTS_0097**	Intracellular growth locus A protein	+	-	-	+	-	-	**AFT92120**	iglA2	20.9/8.82	22.5/8.98	No	Un	cyt	[3,6a]
54,55	**FTS_1747**	Superoxide dismutase	+	+	+	+	+	+	**AFT93366**	sodB	22.0/5.37	22.0/5.39	No	cyt	cyt	[2]

56	**FTS_0224**	Ribosome-recycling factor	+	-	-	-	-	-	**AFT92214**	frr	20.5/5.58	20.5/5.58	No	cyt	cyt	
57,58 59	**FTS_1127**	Intracellular growth locus C protein	+	-	-	+	-	-	**AFT92910**	iglC1	22.1/5.65	22.3/5.65	No	Un	cyt	[2-4,6a,11,12]
57,58 59	**FTS_0099**	Intracellular growth locus C protein	+	-	-	-	-	-	**AFT92122**	IglC2	22.4/5.65	22.6/5.65	No	Un	cyt	[2-4,11,12]
61	**FTS_1275**	Oxidoreductase	+	+	-	+	-	-	**AFT**93017	poxF	27.8/5.35	27.9/5.35	No	cyt	cyt	[2,4]
62,63	**FTS_1414**	Enoyl-[acyl-carrier-protein] reductase [NADH]	+	-	-	-	-	-	**AFT93117**	fabI	27.8/6.22	28.0/6.22	No	Un	cyt	
65	**FTS_0161**	Universal stress protein	+	-	-	-	-	-	**AFT92159**	uspA	30.2/5.78	30.4/5.78	No	Un	cyt	
66,67	**FTS_0222**	Elongation factor Ts	+	-	-	+	-	-	**AFT92212**	tsf	31.0/5.57	31.0/5.57	No	cyt	cyt	[2,4,5,7,12]
68	**FTS_1848**	Glutamine synthetase	+	-	-	-	-	-	**AFT93442**	glnA	38.3/5.33	38.7/5.33	No	cyt	cyt	[7,10]
69,72	**FTS_0479**	Glycine cleavage system aminomethyltransferase	+	-	-	-	-	-	**AFT**92405	gcvT	39.5/5.73	39.6/5.73	No	cyt	cyt	[5,10]
70	**FTS_1118**	Phosphoglycerate kinase	+	-	-	-	-	-	**AFT92904**	pgk	42.0/5.68	42.1/5.68	No	cyt	cyt	
73,74	**FTS_1108**	Beta-ketoacyl-ACP synthase II	+	+	-	+	-	-	**AFT92894**	fabF	44.0/5.62	44.3/5.62	No	cyt	cyt	[3]
76	**FTS_0582**	Acetyl-CoA-acyltransferase	+	-	-	-	-	-	**AFT92483**	fadA	41.7/5.74	42.0/5.74	No	cyt	cyt	[3]
77,78	**FTS_1744**	Citrate synthase	+	-	-	-	-	-	**AFT93363**	gltA	47.4/6.12	47.4/6.12	No	cyt	cyt	[2,4]
79	**FTS_1551**	Acetyl-CoA carboxylase, biotin carboxylase subunit	+	-	-	-	-	-	**AFT93218**	accC	50.0/6.85	50.4/6.85	No	cyt	cyt	
80	**FTS_0292**	Acetyl- CoA carboxylase carboxyl transferase subunit alpha	+	-	-	+	-	-	**AFT92276**	accA	35.4/7.69	35.6/7.68	No	cyt	cyt	[2,4,6a]
82	**FTS_0553**	Two-component response regulator	+	-	-	-	-	-	**AFT92465**	-	25.5/6.01	25.5/6.02	No	cyt	cyt	[2]
83	**FTS_1857**	Cell division protein FtsZ	+	-	-	-	-	-	**AFT93450**	ftsZ	39.7/4.80	39.8/4.76	No	cyt	cyt	[2,5,6,6a,8, 10-12]

a) Accesion number (GenBank) according NCBI database
b) Expasy – prediction of theoretical Mr and pI
c) SignalP program was used for prediction of signal peptide presence

d) PSORTb 3.0.2 program was applied to predict protein function and localization: cyt, cytoplasmic protein; Sp, signal peptide; Un, unknown function and localization; per, periplasmic protein
e) LipoP 1.0 program was used for prediction of lipoproteins: cyt, cytoplasmic protein; SpI, signal peptidase type I; SpII, signal peptidase type II, TMH – n-terminal transmembrane helix

<u>REFERENCES</u>

[1] Havlasová J, Hernychová L, Halada P, Pellantová V, Krejsek J, Stulík J, Macela A, Jungblut PR, Larsson P, Forsman M.: Mapping of immunoreactive antigens of Francisella tularensis live vaccine strain.Proteomics. 2002 Jul;2(7):857-67.

[2] Hubálek M, Hernychová L, Havlasová J, Kasalová I, Neubauerová V, Stulík J, Macela A, Lundqvist M, Larsson P.: Towards proteome database of Francisella tularensis. J Chromatogr B Analyt Technol Biomed Life Sci. 2003 Apr 5;787(1):149-77.

[3] Hubalek M et al.: Comparative proteome analysis of cellular proteins extracted from highly virulent *Francisella tularensis* ssp. *tularensis* and less virulent *F. tularensis* ssp. *holarctica* and *F. tularensis* ssp. *mediaasiatica*. *Proteomics* 2004, 4(10):3048-3060.

[4] Havlasova J et al.: Proteomic analysis of anti-*Francisella tularensis* LVS antibody response in murine model of tularemia. *Proteomics* 2005, 5(8):2090-2103.

[5] Twine SM et al.: Immunoproteomic analysis of the murine antibody response to successful and failed immunization with live anti-*Francisella* vaccines. *Biochemical and biophysical research communications* 2006, 346(3):999-1008.

[6] Janovska S et al.: Proteomic analysis of antibody response in a case of laboratory-acquired infection with *Francisella tularensis* subsp. *tularensis. Folia microbiologica* 2007, 52(2):194-198.
[6a] Janovska S, Pavkova I, Hubalek M, Lenco J, Macela A, Stulik J. Identification of immunoreactive antigens in membrane proteins enriched fraction from Francisella tularensis LVS. Immunol Lett 2007, Feb 15;108(2):151-9. Epub 2007 Jan 10

[7] Eyles JE et al.: Immunodominant *Francisella tularensis* antigens identified using proteome microarray. *Proteomics* 2007, 7(13):2172-2183

[8] Twine SM et al.: Immunoproteomics analysis of the murine antibody response to vaccination with an improved *Francisella tularensi*s live vaccine strain (LVS). *PloS one* 2010, 5(4):e10000.

[9] Sundaresh S, Randall A, Unal B, Petersen JM, Belisle JT, Hartley MG, Duffield M, Titball RW, Davies DH, Felgner PL, Baldi P. From protein microarrays to diagnostic antigen discovery: a study of the pathogen Francisella tularensis. Bioinformatics. 2007 Jul 1;23(13):i508-18.

[10] Fulton KM, Zhao X, Petit MD, Kilmury SL, Wolfraim LA, House RV, Sjostedt A, Twine SM. Immunoproteomic analysis of the human antibody response to natural tularemia infection with Type A or Type B strains or LVS vaccination. Int J Med Microbiol. 2011 Nov;301(7):591-601. doi: 10.1016/j.ijmm.2011.07.002. Epub 2011 Aug 27.

[11] Chandler JC, Sutherland MD, Harton MR, Molins CR, Anderson RV, Heaslip DG, Bosio CM, Belisle JT. Francisella tularensis LVS surface and membrane proteins as targets of effective post-exposure immunization for tularemia. J Proteome Res. 2015 Feb 6;14(2):664-75. doi: 10.1021/pr500628k. Epub 2014 Dec 29.

[12] Gaur R, Alam SI, Kamboj DV. Immunoproteomic Analysis of Antibody Response of Rabbit Host Against Heat-Killed Francisella tularensis Live Vaccine Strain. Curr Microbiol. 2017 Apr;74(4):499-507. doi: 10.1007/s00284-017-1217-y. Epub 2017 Feb 23.

Supplementary Table S2

Gene locus	Gene	Name of protein	Reactivity GF[a]	Reactivity SPF[b]	COGs[c]	Eukarytotic ortholog/analog	Eukaryotic cell compartment
FTS_1275	-	Oxidoreductase	X	X	C	Transmembrane oxidoreductases	Membranes
FTS_1476	*ugpQ*	Glycerophosphoryl diester phosphodiesterase	X	X	C	GDPDL3 (in plant - *Arabidopsis thaliana*)	Cell membrane
FTS_1517	*sucC*	Succinyl-CoA synthetase subunit beta	X	X	C	Succinyl-CoA ligase [ADP-forming] subunit beta, mitochondrial	Mitochondria
FTH_0376	-	Manose-6-phosphate isomerase	X	X	G	Manose-6-phosphate isomerase	Cytosol
FTS_1656	*glpX*	Fructose-1,6-bisphosphatase II	X	X	G	Glycogen synthesis from carbohydrate precursors	Nucleus, Cytosol
FTS_1486	*kbl*	2-amino-3-ketobutyrate coenzyme A ligase	X	X	H	2-amino-3-ketobutyrate coenzyme A ligase	Mitochondria, Nucleus
FTS_1510	*pdxS*	Pyridoxal biosynthesis lyase PdxS	X	X	H	PdxS (missing in mammals)	Cytosol
FTS_0292	*accA*	Acetyl-CoA carboxylase carboxyl transferase subunit alpha	X	X	I	Acetyl-CoA carboxylase	Endoplasmic reticulum
FTS_1108	*fabF*	Beta-ketoacyl-ACP synthase II	X	X	I	3-Oxoacyl-(acyl-carrier protein) synthases	Mitochondria
FTS_0222	*tsf*	Elongation factor Ts	X	X	J	Eukaryotic EF-1B	Mitochondria
FTS_0935	*ychF*	GTP depending nucleic acid-binding protein EngD	X	X	J	Ribosome-binding ATPase YchF ortologs	Nucleus, Cytosol
FTS_1002	*rplI*	50S ribosomal protein L9	X	X	J	50S ribosomal protein L9, chloroplastic (plants)	Ribosomes
FTS_1155	*yhbH*	Sigma 54 modulation protein	X	X	J	No	No
FTS_1702	*rplL*	50S ribosomal protein L7/L12	X	X	J	The L7/L12 ribosomal domain (little homology to eubacterial L7/L12 proteins)	Ribosomes
FTS_1709	*tufA*	Elongation factor Tu	X	X	J	eEF-1 subunit α, Elongation fdactor TU mitochondrial	Cytosol, Mitochondrial envelope, Plasma membrane
FTS_0609	*rho*	Transcription termination factor Rho	X	X	K	No	No
FTS_0882	*tig*	Trigger factor	X	X	O	Ribosome-associated chaperones	Cytosol
FTS_1167	*dnaK*	Chaperon protein DnaK	X	X	O	Eukaryotic hsp70 analogues	Cytosol, Nucleus
FTS_1204	*sufC*	SufS activator complex sufC subunit	X	X	O	Suf system of chloroplasts	Chloroplasts

FTS_1532	*trxB*	Thioredoxin reductase	X	X	O	Thioredoxin reductase	Cytosol, Nucleus
FTS_0617	*bfr*	Bacterioferritin	X	X	P	Ferritin proteins	Cytosol
FTS_1471	*katG*	Peroxidase / catalase	X	X	P	Bifunctional catalase-peroxidases (in lower eukaryotes)	Peroxisome
FTS_1747	*sod B*	Iron/manganese superoxide dismutase family protein	X	X	P	Superoxide dismutase	Mitochondria, Cytosol
FTS_0097	*iglA1*	IglA protein	X	X	S	No	No
FTS_1366	-	Hypothetical protein	X	X	S	No	No
FTS_1795	-	Hypothetical protein	X	X	S	No	No
FTS_0309	*aceE*	Pyruvate dehydrogenase subunit E1	X		C	Pyruvate dehydrogenase E1 component	Mitochondria
FTS_0527	*fumA*	Fumerate hydratase	X		C	Fumerate hydratase	Mitochondria, Cytosol
FTS_1518	*sucD*	Succinyl-CoA synthetase subunit alpha	X		C	Succinyl-CoA ligase [ADP-forming] subunit alpha	Mitochondria
FTS_1744	*gltA*	Citrate synthase	X		C	Citrate synthase	Mitochondria
FTS_1750	*aptpC*	ATP synthase epsilon chain	X		C	ATP synthase epsilon chain	Mitochondria
FTS_1753	*aptA*	ATP synthase subunit alpha	X		C	ATP synthase subunit alpha	Mitochondria
FTS_1857	*ftsZ*	Cell division protein FtsZ	X		D	Tubulin	Cytosol
FTS_0088	*trpB*	Tryptophan synthase subunit beta	X		E	Tryptophan synthase beta chain (it is absent from Animalia)	No
FTS_0479	*gcvT*	Glycine cleavage system	X		E	Aminomethyltransferase	Mitochondria
FTS_1848	*glnA*	Glutamine synthetase	X		E	Glutamine synthetase	Cytosol, Cell membrane, Endoplasmic reticulum
FTS_0787	*adk*	Adenylate kinase	X		F	Adenylate kinase	Cytosol
FTS_1359	*gmk*	Guanylate kinase	X		F	Guanylate kinase	Cytosol
FTS_1432	*deoD*	Purine nucleoside phosphorylase	X		F	Purine nucleoside phosphorylase	Cytosol, Cytoskeleton, Nucleus
FTS_1116	*tktA*	Transketolase	X		G	Transketolase	Cytosol, Nucleus, Peroxisome
FTS_1118	*pgk*	Phosphoglycerate kinase	X		G	Phosphoglycerate kinase	Cytosol, Membrane rafts
FTS_1457	*gpml*	Phosphoglyceromutase	X		G	Phosphoglycerate mutase, 2,3-bisphosphoglycerate-independent	Cytosol, Mitochondrial envelope, Plasma membrane
FTS_1735	*tpiA*	Triosephosphate isomerase	X		G	Triosephosphate isomerase	Cytosol

FTS_0675	*panB*	3-methyl-2-oxobutanoate hydroxymethyltransferase	X		H	3-methyl-2-oxobutanoate hydroxymethyltransferase	Mitochondria
FTS_0582	*fadA*	Acetyl-CoA-acyltransferase	X		I	Acetyl-CoA C-acyltransferase	Cytosol
FTS_1414	*fab1*	Enoyl-[acyl-carrier-protein] reductase	X		I	No	No
FTS_1551	*accC*	Acetyl-CoA carboxylase, biotin carboxylase subunit	X		I	Acetyl-CoA carboxylase	Cytosol
FTS_0178	*efp*	Elongation factor P	X		J	EIF5A	Cytosol
FTS_1552	*accB*	Acetyl-CoA carboxylase, biotin carboxy carrier protein subunit	X		I	Acetyl-CoA carboxylase, biotin carboxy carrier protein subunit	Endoplasmic reticulum (most eukaryotes), Chloroplast
FTS_0224	*frr*	Ribosome-recycling factor	X		J	Ribosome recycling (release) factor (RRF)	Cytosol, Nucleus, Mitochondria
FTS_0351	*rph*	Ribonuclease PH	X		J	RNase PH-like proteins	Cytosol, Nucleus
FTS_1797	*gatA*	Aspartyl/glutamyl-tRNA amidotransferase subunit A	X		J	glutamyl-tRNA(Gln) amidotransferase, A subunit	Mitochondria, Chloroplast
FTS_1862	*rpsA*	30S ribosomal protein S1	X		J	30S ribosomal subunit eukaryotic orthologues	Ribosomes
FTS_0258	*rpoA*	DNA-directed RNA polymerase subunit alpha	X		K	DNA-directed RNA polymerases	Nucleus
FTS_1440	*greA*	Transcription elongation factor GreA	X		K	No	No
FTS_0001	*dnaN*	DNA polymerase III beta subunit	X		L	Proliferating cell nuclear antigen (PCNA)	Nucleus
FTS_0013	*ssb*	Single-stranded DNA-binding protein	X		L	Single-stranded DNA binding protein family (SSBs)	Cytosol, Nucleus
FTS_0008	*ompH*	Outer membrane protein of unknow function	X		M	No	No
FTS_1295	*fopA*	OmpA family protein	X		M	No	No
FTS_0084	*clpB*	Chaperone ClpB	X		O	Caseinolytic peptidase B protein homolog	Mitochondria
FTS_0324	*fkpB*	FKBF-type peptidyl-prolyl cis-trans isomerase	X		O	Prolyl isomerase	Cytosol
FTS_0610	*trx1*	Thioredoxin	X		O	Thioredoxin	Nucleus, Cytosol
FTS_1166	*grpE*	Heat shock protein GrpE	X		O	GrpE protein homolog 1, mitochondrial	Mitochondria
FTS_1670	*groEL*	Chaperonin GroEL	X		O	Hsp60	Mitochondria
FTS_1671	*groES*	Co-chaperonin GroES	X		O	10 kDa chaperonin	Mitochondria
FTS_0836	-	Isochorismatase hydrolase family protein	X		Q	Isochorismatase hydrolases like proteins (fungi and plants)	Cytosol

FTS_0911	*elbB*	Isoprenoid biosynthesis protein	X		Q	Isoprenoid biosynthesis pathway enzymes	Cytoplasm
FTS_0099	*iglC1*	Intracellular growth locus C protein	X		S	No	No
FTS_0815	-	Hypothetical protein	X		S	No	No
FTS_1046	-	Hypothetical protein	X		S	No	No
FTS_1126	*iglB2*	Intracellular growth locus B protein	X		S	No	No
FTS_1201	-	Hypothetical protein	X		S	No	No
FTS_0161	*uspA*	Universal stress protein	X		T	USP can be found in bacteria, archaea, fungi, protozoa and plants	Mitochondria, Chloroplast, Nucleus
FTS_0553	-	Two-component response regulator	X		T	Two component response regulator ("conspicuously absent" from animals)	Nucleus
FTS_0010	*secB1*	Preprotein translocase subunit SecB	X		U	No	No
FTS_0290	*secB2*	Protein-export protein SecB	X		U	No	No
FTS_0387	*purE*	N5-carboxyaminoimidazole ribonucleotide mutase		X	F	No	No
FTS_1120	*fbaA*	Fructose-1,6-bisphosphatase		X	G	Fructose-1,6-bisphosphatase	Cytosol, Nucleus
FTS_1406	*rpe*	D-Ribulose-phosphate 3-epimerase		X	G	No	No
FTS_1877	*purH*	Bifunctional Phosporibosyl-aminoimidazolecarboxamide formyltransferase/IMP cyclo-hydrolase		X	F	No	No
FTS_1120	*fbaA*	Fructose-1,6-bisphosphatase		X	G	Fructose-1,6-bisphosphatase	Cytosol, Nucleus
FTS_0019	*aspS*	Aspartate-tRNA synthase		X	J	Aspartate-tRNA synthetase, type 2	Cytosol
FTS_0877	*miaB*	(Dimethylallyl)adenosine tRNA methylthiotransferase		X	J	No	No
FTS_1818	*pcm*	Protein-L-isoaspartate O-methyltransferase		X	O	Protein-L-isoaspartate(D-aspartate) O-methyltransferase	Cytosol
FTS_0923	-	Hypothetical protein		X	Q	No	No
FTS_1893	-	Putative ABC transporter ATP-binding protein		X	S	No	No
FTS_0889	*hfq*	Host factor I for bacteriophage Q beta replication		X	T	No	No

a) Broadly related immunoreactivity of GF tested sera (regardless of specific reactivity at specific time intervals 12, 24, and 48h.)
b) Broadly related immunoreactivity of SPF tested sera (regardless of specific reactivity at specific time intervals 12, 24, and 48h.)
c) Clusters of Orthologous Groups (COGs) - prediction of function and localization characteristics based on ORFs sequences of identified protein using EggNOG v. 5.0.0
